# Supplementary material for: All-cause and cause-specific mortality among individuals imprisoned for driving under the influence of alcohol and drugs in Norway (2000–2016): a retrospective cohort study
Source: BMJ Open. 2023 Dec 30;13(12):e078848. doi: 10.1136/bmjopen-2023-078848 (PMC10759136; doi:10.1136/bmjopen-2023-078848)
Supplement: Supplementary data [file bmjopen-2023-078848supp001.pdf]

Supplementary tables

Supplementary table 1. Mean age at death (with standard deviation) for all causes of death

Cause of death	No DUI	DUI		
		DUI only	DUI drug	DUI other
Cancer	58.87 (10.93)	62.97 (9.29)	58.90 (10.64)	61.87 (9.23)
Circulatory	56.70 (12.55)	60.80 (10.77)	57.02 (12.24)	59.18 (10.63)
Respiratory	58.99 (11.33)	63.55 (10.43)	60.67 (13.55)	63.50 (12.72)
Digestive	57.08 (10.82)	56.82 (7.59)	54.00 (11.90)	54.00 (8.41)
Alcoholic liver disease	52.12 (7.91)	56.53 (7.24)	54.46(7.92)	52.31 (5.49)
Other internal	51.47 (14.18)	60.25 (11.53)	51.76 (13.74)	60.90 (15.27)
**Total internal causes**	**56.57 (12.23)**	**61.12 (10.20)**	**56.95 (12.13)**	**59.57 (10.93)**
Transport-related	36.62 (11.40)	49.54 (7.98)	38.33 (12.09)	38.47 (12.38)
Intoxication[1]	38.45 (11.38)	48.49 (13.23)	40.27 (12.02)	46.21 (13.23)
Alcohol-related	54.08 (9.53)	57.89 (9.39)	54.96 (11.81)	54.61 (10.25)
Drug-related	36.59 (10.08)	40.56 (10.54)	37.44 (9.80)	39.54 (11.48)
Suicide	37.51 (11.30)	42.78 (14.65)	37.37 (9.75)	41.32 (12.89)
Homicide	36.19 (11.06)	-	41.00 (9.68)	-
Other accident-related	45.14 (14.84)	50.62 (11.91)	47.08 (14.88)	49.97 (15.60)
**Total external causes**	**38.69 (11.93)**	**47.75 (13.32)**	**40.38 (12.22)**	**45.11 (13.88)**
Unexplained	50.94 (13.25)	58.34 (11.99)	53.90 (12.81)	53.00 (12.02)
Unknown cause	47.21 (13.32)	55.72 (12.99)	48.52 (13.55)	54.73 (10.81)
**Total deceased**	**46.42 (14.86)**	**56.39 (13.03)**	**47.62 (14.63)**	**52.77 (14.12)**

[1] Alcohol- and drug-related combined
