# Supplementary material for: All-cause and cause-specific mortality among individuals imprisoned for driving under the influence of alcohol and drugs in Norway (2000–2016): a retrospective cohort study
Source: BMJ Open. 2023 Dec 30;13(12):e078848. doi: 10.1136/bmjopen-2023-078848 (PMC10759136; doi:10.1136/bmjopen-2023-078848)
Supplement: Supplementary data [file bmjopen-2023-078848supp002.pdf]

Table 4. Logistic regression for all-causes, natural causes and unnatural causes of death. Estimates given as crude (cOR) and adjusted odds ratios (aOR) with corresponding 95% confidence intervals. The level of statistical significans was $p \leq 0.05$

	**All cause of death, n=8053**				**Natural causes of death, n=3379**				**Unatural causes of death, n=4139**			
	cOR		aOR[1]		cOR		aOR[1]		cOR		aOR[1]	
Sex (female ref.)	1.21 (1.12-1.31)	**<0.001**	1.22 (1.12-1.32)	**<0.001**	1.20 (1.07-1.36)	**0.003**	1.18 (1.04-1.33)	**0.011**	1.16 (1.04-1.29)	**0.009**	1.11 (1.00-1.24)	**0.063**
Age[2]	1.06 (1.05-1.06)	**<0.001**	1.06 (1.05-1.06)	**<0.001**	1.11 (1.10-1.11)	**<.001**	1.11 (1.10-1.11)	**<.001**	1.01 (1.01-1.02)	**<.001**	1.01 (1.01-1.02)	**<.001**
No. of convictions	1.03 (1.01-1.04)	**<0.001**	0.99 (0.97-1.00)	**0.092**	0.88 (0.86-0.91)	**<0.001**	0.85 (0.83-0.88)	**<0.001**	1.09 (1.08-1.11)	**<0.001**	1.06 (1.04-1.08)	**<0.001**
Conviction (no dui ref.):	-		-		-		-		-		-	
DUI-only	1.28 (1.18-1.38	**<.001**	0.99 (0.91-1.07)	**0.916**	1.91 (1.72-2.11)	**<.001**	1.11 (0.99-1.24)	**0.066**	0.78 (0.69-0.89)	**<.001**	0.78 (0.69-0.89)	**<.001**
DUI-drug	1.59 (1.51-1.69)	**<.001**	1.51 (1.44-1.63)	**<.001**	1.61 (1.47-1.75)	**<.001**	1.80 (1.64-1.98)	**<.001**	1.60 (1.49-1.73)	**<.001**	1.45 (1.34-1.57)	**<.001**
DUI-other	1.42 (1.26-1.59)	**<.001**	1.23 (1.09-1.38)	**<.001**	1.72 (1.46-2.03)	**<.001**	1.34 (1.12-1.60)	**0.001**	1.16 (0.98-1.37)	**0.095**	1.13 (0.95-1.34)	**0.164**

[1]Adjusted for covariates significant in crude analyses
[2]Age at last incarceratinon
