# Supplementary material for: All-cause and cause-specific mortality among individuals imprisoned for driving under the influence of alcohol and drugs in Norway (2000–2016): a retrospective cohort study
Source: BMJ Open. 2023 Dec 30;13(12):e078848. doi: 10.1136/bmjopen-2023-078848 (PMC10759136; doi:10.1136/bmjopen-2023-078848)
Supplement: Author's manuscript [file bmjopen-2023-078848.draft_revisions.pdf]

BMJ Open is committed to open peer review. As part of this commitment we make the peer review history of every article we publish publicly available.

When an article is published we post the peer reviewers' comments and the authors' responses online. We also post the versions of the paper that were used during peer review. These are the versions that the peer review comments apply to.

The versions of the paper that follow are the versions that were submitted during the peer review process. They are not the versions of record or the final published versions. They should not be cited or distributed as the published version of this manuscript.

BMJ Open is an open access journal and the full, final, typeset and author-corrected version of record of the manuscript is available on our site with no access controls, subscription charges or pay-per-view fees (http://bmjopen.bmj.com).

If you have any questions on BMJ Open's open peer review process please email info.bmjopen@bmj.com

BMJ Open

All-cause and cause-specific mortality among individuals imprisoned for Driving under the Influence of alcohol and drugs in Norway (2000-2016)

Journal:	*BMJ Open*
Manuscript ID	bmjopen-2023-078848
Article Type:	Original research
Date Submitted by the Author:	14-Aug-2023

Complete List of Authors:	Jamt, Ragnhild; Oslo University Hospital, Department of Forensic Sciences<br>Bukten, Anne; University of Oslo, Norwegian Centre For Addiction Research; Oslo University Hospital, Section for Clinical Addiction Research<br>Stavseth, Marianne ; Oslo University Hospital, Section for Clinical Addiction Research; University of Oslo, Norwegian Centre for Addiction Research<br>Bogstrand, Stig Tore; Oslo University Hospital, Department of Forensic Sciences; University of Oslo, Department of Public Health Science<br>Tverborgvik, Torill; University of Oslo, Norwegian Centre for Addiction Research

Keywords:	REGISTRIES, EPIDEMIOLOGY, Retrospective Studies

SCHOLARONE™
Manuscripts

I, the Submitting Author has the right to grant and does grant on behalf of all authors of the Work (as defined in the below author licence), an exclusive licence and/or a non-exclusive licence for contributions from authors who are: i) UK Crown employees; ii) where BMJ has agreed a CC-BY licence shall apply, and/or iii) in accordance with the terms applicable for US Federal Government officers or employees acting as part of their official duties; on a worldwide, perpetual, irrevocable, royalty-free basis to BMJ Publishing Group Ltd ("BMJ") its licensees and where the relevant Journal is co-owned by BMJ to the co-owners of the Journal, to publish the Work in this journal and any other BMJ products and to exploit all rights, as set out in our licence.

The Submitting Author accepts and understands that any supply made under these terms is made by BMJ to the Submitting Author unless you are acting as an employee on behalf of your employer or a postgraduate student of an affiliated institution which is paying any applicable article publishing charge ("APC") for Open Access articles. Where the Submitting Author wishes to make the Work available on an Open Access basis (and intends to pay the relevant APC), the terms of reuse of such Open Access shall be governed by a Creative Commons licence – details of these licences and which Creative Commons licence will apply to this Work are set out in our licence referred to above.

Other than as permitted in any relevant BMJ Author's Self Archiving Policies, I confirm this Work has not been accepted for publication elsewhere, is not being considered for publication elsewhere and does not duplicate material already published. I confirm all authors consent to publication of this Work and authorise the granting of this licence.

For peer review only

All-cause and cause-specific mortality among individuals imprisoned for Driving under the Influence of alcohol and drugs in Norway (2000-2016)

Ragnhild E. G. Jamt[1], Anne Bukten[2,3,4], Marianne Riksheim Stavseth[2,3], Stig T. Bogstrand[1,5], Torill Tverborgvik[2]

[1]Department of Forensic Sciences, Oslo University Hospital, Oslo, Norway

[2]Norwegian Centre for Addiction Research, University of Oslo, Oslo, Norway

[3]Section for Clinical Addiction Research, Oslo University Hospital, Oslo, Norway

[4]University College of Norwegian Correctional Service, Lillestrøm, Norway

[5]Department of Public Health Science, University of Oslo, Oslo, Norway

Corresponding author:

Ragnhild E. G. Jamt

Postal address: Oslo University Hospital, Department of Forensic Sciences,

Post box 4950 Nydalen, 0424 Oslo, Norway

E-mail address: rmraej@ous-hf.no

For peer review only

Abstract

Objectives:

To describe all-cause and cause-specific mortality and to investigate factors associated with mortality among individuals imprisoned for driving under the influence (DUI) of alcohol and psychoactive drugs in the Norwegian prison population.

Design:

Retrospective cohort study. The Norwegian prison registry was linked to the Norwegian Cause of Death Registry (2000-2016).

Setting:

Norway

Participants:

The cohort consisted of 96 856 individuals imprisoned in Norway over a 17-year period (January 1st, 2000, until December 31st, 2016) obtained from the Norwegian prison registry.

Primary and secondary outcome measures:

Adjusted odds ratios (aOR) were calculated for death due to any, internal and external causes of death. Analyses were stratified according to DUI convictions: no DUI convictions, only DUI convictions, DUI and at least one other drug and alcohol conviction, and DUI and at least one conviction other than drug and alcohol.

Results:

In total, 29.3% of the cohort had one or more imprisonments for DUI during the observation period. Heterogeneity within the DUI-group was identified in terms of risk of death. The risk of all-cause mortality was elevated for those convicted of DUI, but only in combination with other types of crimes. The risk of death from internal causes was significantly elevated for all individuals convicted of DUI. The risk of death from external causes was lower for those convicted of DUI only (aOR = 0.8, 95% confidence interval (CI) = 0.7-0.9) and elevated for those with DUI-convictions in combination with other drug and alcohol convictions (aOR = 1.6, 95% CI = 1.5-1.7).

For peer review only - http://bmjopen.bmj.com/site/about/guidelines.xhtml

Conclusions:

The risk of all-cause mortality was significantly elevated for those convicted for DUI, but only in combination with other types of crimes, and especially in combination with drug and alcohol crime.

Strengths and limitations:

- We were able to study the mortality of the entire Norwegian prison population through linkage of mandatory national registries during an observation period of 17 years.

- Linkage of datasets through unique PINs reduces the chances of linkage bias and loss to follow up.

- The lack of information on background socio-demographics and health variables in the dataset implies that we could not adjust for important pre-existing conditions associated with mortality.

- Data on individuals who served conditional sentences outside prison for DUI was not available.

For peer review only

For peer review only - http://bmjopen.bmj.com/site/about/guidelines.xhtml

INTRODUCTION

The literature on driving under the influence of psychoactive substances (alcohol, psychoactive medicinal drugs, and illicit drugs; DUI) has mainly revolved around topics such as the prevalence of DUI and the risks associated with road traffic injuries and deaths (1-5). DUI poses a threat to the lives and safety of people who engage in DUI (6), as well as other road users (7); however, the research regarding future outcomes of people driving under the influence of alcohol or drugs is both limited and somewhat outdated (8, 9).

Drivers who engage in DUI have been characterized by serious health problems such as substance use and dependence, major depressive disorders, anxiety disorders, hyperactivity disorders and risk-taking dispositions (8). People arrested for DUI exhibit similar patterns of health problems and mortality as found among people with alcohol use disorders (10). Studies by Gjerde et al. and Ruud et al. found that between 35-60% of those arrested for DUI in Norway had an alcohol use disorder (11-13). In Germany, more than 80% of persons arrested for DUI of alcohol had a blood alcohol concentration (BAC) higher than 1.9 g/dl and could be characterized as having alcohol use disorders (14). People convicted of DUI have also been characterized by socially disadvantaged backgrounds in terms of lower levels of education and higher rates of unemployment, disability pensions and divorce compared to the general population (15).

The most recent roadside surveys in Norway found the total prevalence of alcohol and psychoactive drugs among random drivers to be around 5% (16). While the prevalence of drivers with a BAC above the legal limit of 0.02 g/dl was 0.2%, the prevalence of psychoactive medicinal drugs and of illicit drugs were 3.0% and 1.7%, respectively. Hence, driving under the influence of alcohol is less common in Norway. Legislation on DUI, including strong law enforcement and strict punishments over many years is likely to have contributed to the low numbers of DUI in Norway (17). However, the prevalence of psychoactive substances among drivers arrested on suspicion of DUI and among drivers involved in road traffic crashes is high (18, 19). A paper reviewing the risk of serious road traffic crashes while engaging in DUI found higher risks in Norwegian and Finnish studies than in studies from other countries (20). It has been suggested that in countries where DUI is considered socially unacceptable, people driving under the influence are a more selected group with higher incidence of substance use disorders (SUDs) along with higher levels of sensation-

For peer review only - http://bmjopen.bmj.com/site/about/guidelines.xhtml

seeking and risk-taking behavior (21). It is furthermore suggested that people engaging in risky driving behavior might be less affected by threats of punishment (21).

In 2019, 21% of all prison sentences in Norway included at least one DUI-offence (22). Several studies have demonstrated that the prison population has a greater risk of premature death compared to the general population, particularly in the short-term period following release from prison (23-26). A study based on the Norwegian police registration system showed that the risk of premature death is particularly high among individuals arrested for alcohol and drug related crimes, compared to both the general population and individuals arrested for other criminal offences (9).

Although there are similarities between people convicted of DUI and individuals with alcohol use disorders in terms of health problems, the entire group of people convicted of DUI is large and heterogeneous, and therefore more specific knowledge on the risk factors and mortality has been warranted for this group (10).

Using data on all individuals imprisoned in Norway in the period 2000-2016, we defined three sub-categories of the DUI group in order to examine combinations of DUI convictions and other convictions in relation to mortality: *DUI-only* as those having no other convictions but DUIs during the observation period; *DUI-drug* as those having both DUI and at least one other drug and alcohol conviction during the observation period; and *DUI-other* as those having DUI and at least one other conviction other than drug and alcohol conviction during the observation period. The aims of this paper were to:

1. describe the different groups of individuals imprisoned for DUI in the Norwegian prison population

2. describe all-cause and cause-specific mortality in the DUI-groups and compare with individuals imprisoned for other crimes than DUI, and

3. investigate factors associated with internal and external mortality in the prison population.

For peer review only

For peer review only - http://bmjopen.bmj.com/site/about/guidelines.xhtml

METHODS

Design and study population

In this retrospective cohort study, we used data from the Norwegian Prison Release Study (nPRIS) (27), including all persons imprisoned in Norway over a 17-year period (January 1st 2000, until December 31st 2016). The nPRIS-cohort constitutes 114 745 individuals collected from the Norwegian Prison Registry. The data were linked to the Norwegian Cause of Death Register (NCoDR) through the unique 11-digit Personal Identification Number (PIN) given to persons who are born in Norway, have a valid residence permit for at least six months or have officially moved to Norway. In total, 19 140 individuals were excluded from the study cohort due to lack of a valid PIN. Hence, the study population consisted of 96 856 individuals contributing to 167 068 releases.

Setting

Norway has practiced a per se law for driving under the influence of alcohol since 1936. Graded sanctioning is practiced; a driver is sentenced to conditioned imprisonment for driving with BACs between 0.05g/dl and 0.12 g/dl, and unconditioned imprisonment for BACs above 0.12 g/dl (28). *Per se* limits for 28 psychoactive drugs were implemented in 2012/2016 corresponding to a BAC of 0.02 g/dl (29). In addition, limits for graded sanction corresponding to BACs of 0.05 and 1.2 g/dl for 24 of these drugs have been implemented (30).

Norway is characterized by having low rates of imprisonment. In 2021, the prison population rate per 100 000 of the national population was 56 in Norway, compared to 629 in the US and 131 in the UK (31). The Norwegian penal system aim at rehabilitation in that the sentence is recognized as the punishment, and universal health care, including drug treatment, is to be provided in prison as it is outside of prison (32).

Norwegian prison units are public and are located all over Norway. This prison organization allows most prisoners to preserve geographical closeness to friends and family. In 2020, 18% of all unconditional sentences lasted less than 30 days and 76% lasted up to one year (33). Women constitute a minority in Norwegian prisons, with an annual proportion of approximately 5% (34).

Data sources

The Norwegian Prison Registry serves administrative and statistical purposes and includes personal data on all persons imprisoned in Norway, including age, sex, convictions, and sentences (32). The

For peer review only - http://bmjopen.bmj.com/site/about/guidelines.xhtml

registry also includes date of admission and date of release, both for sentences served and time spent on remand.

The Norwegian Cause of Death Registry (NCoDR) includes death certificates reported by medical doctors after examination of the deceased. The NCoDR includes information on time of death along with the underlying cause of death (the disease or injury which initiated the chain of morbid events leading directly to death) and the immediate causes of death (the terminal event or complication present at the time of death) (35). All causes of death are coded using the International Classification of Diseases, 10th revision (ICD-10) (36).

Measures

We categorized all causes of death as either internal or external, as described in Table 1. Internal causes were defined as ICD-10 chapters A through Q, while external causes were defined as ICD-10 chapters V through Y. Drug- and alcohol-related causes (F10-F19) were defined as external causes of death (Table 1).

We classified types of convictions according to the nine-group classification used for Statistics Norway's official crime statistics (37). In their statistics, DUI offences are included in the category "Drug and alcohol offences", but as we wanted to study this offence in particular, we constructed a subgroup with DUI convictions exclusively. Thus, the ten-group classification used in this paper were: Property theft; Other offences for profit; Criminal damage; Drug and alcohol offences (not including DUI); Public order and integrity violations; Sexual offences; Traffic offences; Violence and maltreatment; Other offences; and DUI. One imprisonment can include multiple convictions. Convictions were aggregated across the study period, and dummy coded as "convicted of" (1) or "not convicted of" (0).

For peer review only

Table 1. Categorization of causes of death according to ICD-10 codes.

**Cause of death**	**ICD-10 code**
Cancer	C00-C97
Circulatory	I00-I99
Respiratory	J00-J99
Digestive	K00-K69, K71-K93
Alcoholic liver disease	K70
Other internal	A,B,D-H,L-Q
Internal causes of death	All codes listed above
Transport-related	V, Y85
Intoxication[1]	
Alcohol-related	F10,X45,Y15
Drug-related	F11,F12,F14-16,F19, X41,X42,X44, Y11,Y12,Y14
Suicide	X60-X84
Homicide	X85-X99, Y00-Y09
Other accident-related	W,X2,Y2
External causes of death	All codes listed above (excluding internal causes)
Unexplained	R95-R99
Unknown cause	No ICD-10 code recorded in NDR

[1] Alcohol- and drug-related causes combined

Ethics

The study was approved by the Regional Committees for Medical and Health Research Ethics, Region South-East Norway, reference number 2012/140 and the Norwegian Directorate for Correctional Services. The linkage was performed by the Norwegian Institute of Public Health, who prepared the files for analysis.

Statistical analyses

All analyses were performed using SPSS, version 26. We made group comparisons using Student's t-test or Mann-Whitney U-test for continuous data and X^2-test for categorical data.

The associations between mortality and DUI convictions were examined using logistic regression. Individuals who died due to internal causes differed significantly from those who died due to external causes. Therefore, separate logistic regression models were fitted. The three regression models were defined with the following outcome of interest 1) all-cause mortality (n=8053) 2) death due to internal causes (n=3379), and 3) death due to external causes (n=4139). To avoid competing risks, unknown and external cause deaths were excluded from analysis in the second regression model, and unknown and internal cause deaths were excluded from analysis in the third

For peer review only - http://bmjopen.bmj.com/site/about/guidelines.xhtml

model. Univariate regression models were fitted, and statistically significant covariates (p-value < 0.05) were included in the multivariable analysis.

Patient and public involvement

Patients and/or the public were not involved in the design, or conduct, or reporting, or dissemination plans of this research.

RESULTS

Participants

The cohort consisted of 96 856 individuals of which 28 393 individuals (29.3%) were convicted of any DUI (Table 2). Among these, 8898 (9.2%) were only convicted of DUI (DUI-only), 16 159 (16.7%) were convicted of DUI and at least one drug and alcohol offence (DUI-drug), and 3336 (3.4%) were convicted of DUI and at least one crime other than drug or alcohol related offences (DUI-other) during the observation period (Table 2). The DUI-only group had the highest proportion of women (13.7%) and were older at last incarceration (mean age 38.4 years) compared to the rest.

Approximately one third of the total cohort (n = 30 506) had more than one imprisonment during the observation period, but while this was true for almost 60% of the DUI-drug group, only 5.5% of the DUI-only group had multiple incarcerations during the study period. The overall proportion of deceased among those convicted of any DUI was 10.5% compared to 7.4% among the non-DUI-offenders. The proportion of deceased was highest in the DUI-drug group (11.3%) (Table 2).

For peer review only - http://bmjopen.bmj.com/site/about/guidelines.xhtml

Table 2. Background characteristics of the study population (n=96 856), 1st January 2000 – 31st December 2016.

	No DUI		DUI					
			DUI only		DUI drug		DUI other	
	n	%	n	%	n	%	n	%
Total	68463	70.7	8898	9.2	16159	16.7	3336	3.4
Sex[1]								
Men	61171	89.3	7680	86.3	14870	92.0	3155	94.6
Women	7275	10.6	1218	13.7	1289	8.0	181	5.4
Age at last incarceration[2,3]	34.6 (11.8)		38.4 (14.1)		36.2 (11.4)		36.6 (13.4)	
Number of incarcerations[3,4]	1.6 (1.4)		1.1 (0.3)		2.7 (2.6)		1.6 (0.9)	
Convictions[5]								
Property theft	9974	14.6	-	-	5063	31.3	351	10.5
Other offences for profit	16335	23.9	-	-	4197	26.0	358	10.7
Criminal damage	4045	5.9	-	-	2102	13.0	171	5.1
Drug and alcohol offences[6]	28505	41.6	-	-	16159	100	-	-
Public order/integrity	15379	22.5	-	-	5673	35.1	652	19.5
Sexual offences	5362	7.8	-	-	333	2.1	124	3.7
Traffic offences	3648	5.3	-	-	5372	33.2	1932	57.9
Violence and maltreatment	25597	37.4	-	-	4932	30.8	1061	31.8
Other offences	2278	3.3	-	-	947	5.9	89	2.7
Driving under the influence	-	-	8898	100	16159	100	3336	100
1DUI conviction	-	-	*8657*	*97.3*	*12755*	*78.9*	*3032*	*90.9*
2+DUI convictions	-	-	*241*	*2.7*	*3404*	*21.1*	*304*	*9.1*
Deceased	5064	7.4	825	9.3	1825	11.3	339	10.2

[1] 17 persons missing information on sex
[2] 42 persons missing information on age
[3] Continuous measures presented with mean (SD)
[4] During the study period
[5] During the study period. Note: A person may have several convictions per incarceration and several incarcerations
[6] Not including DUI

Causes of death

Deaths among those convicted of DUI-only were mostly due to internal causes (58.9%; Table 3). Diseases of the circulatory system and cancer were the predominant internal causes of death in all groups. A higher proportion of death due to alcoholic liver disease was found among those convicted of DUI-only (5.2%). Those convicted of DUI-only had the lowest proportion of external causes of death (33.0%).

For peer review only - http://bmjopen.bmj.com/site/about/guidelines.xhtml

Deaths among those convicted of DUI-drug were more often due to external causes (54.1%), as was the case among those convicted of other crimes than DUI (53.9%). Intoxication (drug or alcohol related) accounted for the largest proportion of the external causes of death in all groups. Among those convicted of DUI-drug, the vast majority of the deaths caused by intoxications were drug-related.

About 5% of mortalities in the DUI-drug and DUI-other groups were transport related. The proportion in the DUI-only group was 1.6%.

The DUI-only group was in general older at time of death then the other groups. Mean age at death was 61 years among DUI-only, but only 48 years in the DUI-drug group (Supplementary Table 1). Overall, the non-DUI-offenders had the lowest mean age at death of 46 years.

Table 3. Cause of death (n, %) stratified by DUI-groups.

	**No DUI**		**DUI**					
			**DUI only**		**DUI drug**		**DUI other**	
**Cause of death**	n	%	n	%	n	%	n	%
Cancer	640	12.6	144	17.5	214	11.7	46	13.6
Circulatory	620	12.2	158	19.1	234	12.8	62	18.3
Respiratory	215	4.2	55	6.7	86	4.7	12	3.5
Digestive	98	1.9	22	2.7	43	2.4	7	2.1
Alcoholic liver disease	105	2.1	43	5.2	56	3.1	16	4.7
Other internal	325	6.4	64	7.8	94	5.2	20	5.9
**Total Internal cause**	**2003**	**39.6**	**486**	**58.9**	**727**	**39.8**	**163**	**48.1**
Transport-related	224	4.4	13	1.6	90	4.9	17	5.0
Intoxication[1]	1606	31.8	164	19.9	601	32.9	70	20.6
Alcohol-related	*170*	*3.4*	*75*	*9.1*	*97*	*5.3*	*31*	*9.1*
Drug-related	*1436*	*28.4*	*89*	*10.8*	*504*	*27.6*	*39*	*11.5*
Suicide	569	11.2	54	6.5	160	8.8	28	8.3
Homicide	69	1.4	-	-	31	1.7	-	-
Other accident-related	262	5.2	39	4.7	106	5.8	31	9.1
**Total external cause**	**2730**	**53.9**	**272**	**33.0**	**988**	**54.1**	**149**	**44.0**
Unexplained	140	2.8	35	4.2	52	2.8	12	3.5
Unknown cause	191	3.8	32	3.9	58	3.2	15	4.4
**Total deceased**	**5064**	**100**	**825**	**100**	**1825**	**100**	**339**	**100**

[1] Alcohol- and drug-related combined
- Cells with n < 5 are not displayed

For peer review only - http://bmjopen.bmj.com/site/about/guidelines.xhtml

Factors associated with mortality

After adjusting for relevant covariates, being male and increasing age at last incarceration were factors significantly associated with elevated risk of death from all causes: Sex: (aOR: 1.2, CI: 1.1-1.3); Age: (aOR: 1.1, CI: 1.1-1.1). This was also the case for internal causes: Sex: (aOR: 1.1, CI: 1.0-1.3); Age: (aOR: 1.1, CI: 1.1-1.1), and external causes: Sex: (aOR: 1.1, CI: 1.0-1.3); Age: (aOR: 1.0, CI: 1.0-1.0) (Table 4).

Being convicted of DUI and other crimes significantly elevates the risk of death from all causes (DUI-drug: aOR 1.5, CI: 1.4-1.6, DUI-other: aOR 1.2, CI: 1.1-1.4). For internal causes, being convicted of DUI, either alone (DUI-only: aOR 1.2, CI: 1.0-1.3) or with other crimes (DUI-drug: aOR 1.5, CI: 1.4-1.7; DUI-other: aOR 1.3, CI: 1.1-1.6) was associated with an elevated risk. While being convicted of DUI and one or more drug and alcohol related crimes increased the risk of dying from external causes (aOR 1.6, CI: 1.5-1.7), being convicted of DUI only had a significant protective effect on the risk of dying from external causes (aOR 0.8, CI: 0.7-0.9)

For peer review only

For peer review only - http://bmjopen.bmj.com/site/about/guidelines.xhtml

Table 4. Logistic regression for all-causes, internal causes and external causes of death, crude and adjusted. Estimates gives as crude and adjusted odds ratio's with corresponding 95% confidence intervals.

	**All cause of death n=8053**				**Internal cause of death n=3379**				**External cause of death n=4139**			
	**Crude estimates**		**Adjusted estimates[1]**		**Crude estimates**		**Adjusted estimates[1]**		**Crude estimates**		**Adjusted estimates[1]**	
Sex (female ref.)	1.21 (1.12-1.31)	**<.001**	1.21 (1.12-1.32)	**<.001**	1.20 (1.07-1.36)	**0.003**	1.14 (1.00-1.29)	**0.046**	1.16 (1.04-1.29)	**0.009**	1.13 (1.02-1.26)	**0.023**
Age[2]	1.06 (1.05-1.06)	**<.001**	1.06 (1.05-1.06)	**<.001**	1.11 (1.10-1.11)	**<.001**	1.11 (1.10-1.11)	**<.001**	1.01 (1.01-1.02)	**<.001**	1.01 (1.01-1.02)	**<.001**
Convictions:												
No DUI (ref.)	-	**<.001**	-	**<.001**	-	**<.001**	-	**<.001**	-	**<.001**	-	**<.001**
DUI only	1.28 (1.18-1.38)	**<.001**	1.00 (0.92-1.08)	0.916	1.91 (1.72-2.11)	**<.001**	1.17 (1.05-1.31)	**0.005**	0.78 (0.69-0.89)	**<.001**	0.75 (0.66-0.85)	**<.001**
DUI drug	1.59 (1.51-1.69)	**<.001**	1.51 (1.42-1.60)	**<.001**	1.61 (1.47-1.75)	**<.001**	1.54 (1.41-1.69)	**<.001**	1.60 (1.49-1.73)	**<.001**	1.57 (1.46-1.70)	**<.001**
DUI other	1.42 (1.26-1.59)	**<.001**	1.23 (1.09-1.38)	**<.001**	1.72 (1.46-2.03)	**<.001**	1.30 (1.09-1.55)	**0.003**	1.16 (0.98-1.37)	0.095	1.12 (0.95-1.33)	0.175

[1]Adjusted for covariates significant in crude analyses
[2]Age at last incarceration

For peer review only - http://bmjopen.bmj.com/site/about/guidelines.xhtml

For peer review only

DISCUSSION

In this retrospective cohort study of mortality among people imprisoned for DUI in Norway, we found the risk of all-cause mortality to be significantly elevated for those convicted of DUI, but only in combination with other types of crimes, and especially in combination with other drug and alcohol offences. Being convicted of no other crime than DUI had a protective effect on external cause of death after adjusting for age and sex.

Psychiatric disorders and SUDs are highly prevalent in the prison population (38, 39), and it is reasonable to assume that crimes such as DUI and other drug and alcohol offences could be considered a proxy for SUDs. In our study, almost half of the total cohort had one or more drug and alcohol conviction and 30% had at least one DUI conviction. Among those convicted of DUI higher proportions died from causes typically associated with excessive use of alcohol or drugs, further supporting the assumption that DUI conviction can be a proxy for SUDs. This is in line with previous research findings that the most dominant causes of death among people convicted of DUI are alcohol and drug poisoning/overdose, external causes in terms of accidents and suicide and diseases of the circulatory system (10, 40). In the present study, individuals with a history of both DUI and other drug and alcohol convictions had the highest risk of mortality due to external causes, with the most common causes of death being intoxication and suicide.

Impinen et al., suggests that an over-representation of external causes of death might be an indicator of a risk-taking lifestyle. Sensation-seeking behavior is a personality trait that has been associated with engaging in unusual and risky acts, fast and reckless driving, and alcohol and drug use (41, 42). Sensation-seeking behavior has also been shown to decline with age and is usually more pronounced among males (43). In the present study, those with a history of both DUI and other drug and alcohol convictions were on average the youngest of the DUI-groups when they died, primarily from external causes. For those convicted only of DUI offences, the case was the opposite; they were on average the oldest when they died, primarily from internal causes, and had the largest proportion of females.

The mean age at death was low for the whole cohort and particularly low for death due to external causes. As expected, age was significantly associated with increased risk of death in all models. However, when stratifying on cause of death (internal and external causes), the effect of increasing

For peer review only - http://bmjopen.bmj.com/site/about/guidelines.xhtml

age at last incarceration became more pronounced for the risk of death from internal causes. For every one-year increase in the age at last incarceration the risk of death from internal causes increased by 11%, whereas we observed only 1% increase for external causes. In a study by Nordentoft et al. (44) high excess mortality due to somatic illnesses was found among patients with psychiatric disorders. The life expectancy for patients hospitalized due to psychiatric disorders was 20 years shorter for men and 15 years shorter for women compared to the general population. The excess mortality was highest among patients with SUDs.

Many DUI-offenders also have a history of convictions for other types of crime in addition to the DUI-offence, and the tendency to relapse into DUI is well known (45-47). The results of the present study also show this to be the case. Those convicted of both DUI and other drug and alcohol offences had on average almost three incarcerations during the observation period, and about 40% had two or more convictions for DUI. Among those only convicted of DUI, three percent had more than one conviction.

It is evident from the present study that people convicted of DUI are a heterogenic group with those imprisoned for DUI only standing out from both the other DUI groups and from those imprisoned for other crimes than DUI. We found that the “DUI only”-group had fewer incarcerations, higher mean age at last incarceration, higher proportion of women, and higher prevalence of causes of death related to excessive alcohol consumption. It could be that imprisonment of only DUI is indicative of severe alcohol use, and that this is a group in need of other interventions than people incarcerated for other crimes.

A large proportion of people imprisoned for DUI are likely to have some degree of SUD and/or mental health problems (8), which in turn increases their risk of premature death both from internal and external causes. This suggest that the prison context may offer an important opportunity for interventions in terms of SUD treatment. A Norwegian study on DUI among patients in opioid agonist treatment (OAT) found a 40% reduction in DUI convictions while in treatment. However, patients with two or more convictions for DUI before entering treatment had higher odds for further convictions of DUI during OAT compared with patients having no DUI convictions during the pre-treatment period (48). Multiple DUI convictions might thus be viewed as a proxy for the severity of their substance use.

For peer review only - http://bmjopen.bmj.com/site/about/guidelines.xhtml

Attempting to combat DUI recidivism and to lower the social and economic costs of incarceration various DUI prevention programs have been developed internationally. An example is the *Intensive supervision programs* (ISPs) (49). These programs include home confinement with activities such as screening and assessment of offenders' substance abuse problems, treatment and education related to DUI behavior, electronic monitoring of offender movements and use of ignition interlock devices or other restrictions on driving. At present, the assessment of the effectiveness of DUI prevention programs is inconclusive. However, there is some evidence to support the effectiveness of programs utilizing intensive supervision and education (50). In Norway, the program consists of education, individual- and group therapy sessions and investigations of the offenders' treatment needs, but is only available for individuals serving conditional sentences, and thus disqualifying people with more severe SUDs typically serving unconditional sentences.

The results of the present study should be interpreted with certain limitations in mind. The lack of information on background socio-demographics and health variables in the dataset implies that we could not adjust for important pre-existing conditions and factors associated with mortality. Ideally, a control group for comparison in this study would have been a group of individuals convicted of DUI who had been sentenced to conditional prison. However, data on individuals who served conditional sentences was not available for the present study. A strength of this study is that we were able to study the mortality of the entire Norwegian prison population through linkage of mandatory national registries during an observation period of 17 years. The linkage of datasets through unique PINs reduces the chances of linkage bias and loss to follow up is rare. Furthermore, the classification of all deaths is in accordance with the ICD-10 criteria and cause of death are reported by a clinician according to individual ICD codes, reducing the chances of information bias. The NCoDR is a reliable source of data on causes of death with combined degree of coverage and completeness of 98% of the deaths in Norway (35).

Systematic screening with respect drug and alcohol use disorders is a critically important first step to identify individuals who should be offered treatment during incarceration. However, validated screening tools are scarcely implemented in European prisons (51). The results from our study imply that in places where systematic screening is not routinely carried out data on current and prior offence(s) a person is sentenced to prison for (e.g., DUI, other alcohol and drug offences) might serve as an indicator of harmful alcohol and drug use. This data is readily available to the

For peer review only - http://bmjopen.bmj.com/site/about/guidelines.xhtml

correctional service staff, and can be used as means for further investigations using validated screening tools to properly identify people with potential harmful alcohol and drug use. Moreover, when risk has been identified, provision of evidence-based treatment and harm reduction services must be provided for those in need.

Word count: 3450

For peer review only

For peer review only - http://bmjopen.bmj.com/site/about/guidelines.xhtml

Acknowledgments: We thank the Directorate of the Norwegian Correctional Services and the Norwegian Cause of Death Registry for valuable assistance regarding registry data.

Competing interests: none declared.

Funding: This work was supported by the South-Eastern Norway Regional Health Authority: grant number: 2019091 and the Norwegian Research Council: grant number: 301535.

Contributors: R. E. G. Jamt: Conceptualization, Formal analysis, Investigation, Methodology, Writing – original draft A. Bukten: Conceptualization, Investigation, Methodology, Supervision, Writing – review & editing M. R. Stavseth: Conceptualization, Formal analysis, Investigation, Methodology, Supervision, Writing – review & editing S. T. Bogstrand: Conceptualization, Methodology, Supervision, Writing – review & editing T. Tverborgvik: Conceptualization, Formal analysis, Methodology, Supervision, Writing – review & editing. All authors have approved the final article.

Patient consent for publication: not required.

Data availability: This population study was based on individual-level data from The Norwegian Prison Registry (held by the Directorate of Norwegian Correctional Service) and the Norwegian Cause of Death Register (held by the Norwegian Institute of Public Health). The ethical approval of this research project does not include permission to publicly share the raw data.

For peer review only

References

1. Valen A, Bogstrand ST, Vindenes V, *et al*. Driver-related risk factors of fatal road traffic crashes associated with alcohol or drug impairment. Accid Anal Prev. 2019;131:191-9.
2. Walsh JM, Flegel R, Atkins R, *et al*. Drug and alcohol use among drivers admitted to a Level-1 trauma center. Accid Anal Prev. 2005;37(5):894-901.
3. Bogstrand ST, Larsson M, Holtan A, *et al*. Associations between driving under the influence of alcohol or drugs, speeding and seatbelt use among fatally injured car drivers in Norway. Accid Anal Prev. 2015;78:14-9.
4. Bernhoft IM, Hels T, Lyckegaard A, *et al*. Prevalence and Risk of Injury in Europe by Driving with Alcohol, Illicit Drugs and Medicines. Procedia Soc Behav Sci. 2012;48:2907-16.
5. Christophersen AS, Mørland J, Stewart K, *et al*. International trends in alcohol and drug use among vehicle drivers. Forensic Sci Rev. 2016;28(1):37-66.
6. Jamt REG, Gjerde H, Romeo G, *et al*. Association between alcohol and drug use and arrest for driving under the influence after crash involvement in a rural area of Norway: a case-control study. BMJ Open. 2019;9(1):e023563.
7. Oksanen A, Aaltonen M, Kivivuori J. Driving under the influence as a turning point? A register-based study on financial and social consequences among first-time male offenders. Addiction. 2015;110(3):471-8.
8. Skurtveit S, Christophersen AS, Grung M, *et al*. Increased mortality among previously apprehended drunken and drugged drivers. Drug Alcohol Depend. 2002;68(2):143-50.
9. Skardhamar T, Skirbekk V. Relative Mortality among Criminals in Norway and the Relation to Drug and Alcohol Related Offenses. PloS One. 2013;8(11):e78893.
10. Impinen A, Mäkelä P, Karjalainen K, *et al*. High mortality among people suspected of drunk-driving. An 18-year register-based follow-up. Drug Alcohol Depend. 2010;110(1-2):80-4.
11. Gjerde H, Mørland J. Concentrations of carbohydrate-deficient transferrin in dialysed plasma from drunken drivers. Alcohol Alcohol. 1987;22(3):271-6.
12. Gjerde H, Sakshaug J, Mørland J. Heavy Drinking among Norwegian Male Drunken Drivers: A Study of γ-Glutamyltransferase. Alcohol Clin Exp Res. 1986;10(2):209-12.
13. Ruud J, Gjerde H, Mørland J. Alcohol consumption among convicted drunken drivers in Norway. J Traffic Med. 1993;21(3):121-5.
14. Brinkmann B, Beike J, Köhler H, *et al*. Incidence of alcohol dependence among drunken drivers. Drug Alcohol Depend. 2002;66(1):7-10.
15. Impinen A, Mäkelä P, Karjalainen K, *et al*. The Association between Social Determinants and Drunken Driving: A 15-Year Register-based Study of 81,125 Suspects. Alcohol Alcohol. 2011;46(6):721-8.
16. Furuhaugen H, Jamt REG, Nilsson G, *et al*. Roadside survey of alcohol and drug use among Norwegian drivers in 2016-17: a follow up of the 2008-9 survey. Traffic Inj Prev. 2018:1-32.
17. Gjerde H, Christophersen AS, Normann PT, *et al*. Norwegian roadside survey of alcohol and drug use by drivers (2008-2009). Traffic Inj Prev. 2013;14(5):443-52.
18. Gjerde H, Frost J. Prevalence of alcohol and drugs among drivers killed in road traffic crashes in Norway during 2011–2020. Traffic Inj Prev. 2023:1-6.
19. Valen A, Bogstrand ST, Vindenes V, *et al*. Toxicological findings in suspected drug-impaired drivers in Norway — Trends during 1990–2015. Forensic Sci Int. 2017;280:15-24.
20. Gjerde H, Bogstrand ST, Lillsunde P. Commentary: Why Is the Odds Ratio for Involvement in Serious Road Traffic Accident Among Drunk Drivers in Norway and Finland Higher Than in Other Countries? Traffic Inj Prev. 2014;15(1):1-5.

For peer review only - http://bmjopen.bmj.com/site/about/guidelines.xhtml

21. Ulleberg P. Personality subtypes of young drivers. Relationship to risk-taking preferences, accident involvement, and response to a traffic safety campaign. Transp Res Part F Traffic Psychol Behav. 2001;4(4):279-97.
22. Bukten A, Lokdam NT, Clausen T, *et al*. Use and possession of illegal substances and driving while intoxicated among Norwegian prison inmates 2000-2019. Norwegian Centre for Addiction Research; 2021. Available at: seraf-rapport-3-2021-bruk-besittelse-og-ruspavirket-kjoring-.pdf (uio.no).
23. Binswanger IA, Stern MF, Deyo RA, *et al*. Release from prison--a high risk of death for former inmates. N Engl J Med. 2007;356(2):157-65.
24. Graham L, Fischbacher CM, Stockton D, *et al*. Understanding extreme mortality among prisoners: a national cohort study in Scotland using data linkage. Eur J Public Health. 2015;25(5):879-85.
25. Zlodre J, Fazel S. All-cause and external mortality in released prisoners: systematic review and meta-analysis. Am J Public Health. 2012;102(12):e67-75.
26. Kariminia A, Butler T, Corben S, *et al*. Extreme cause-specific mortality in a cohort of adult prisoners—1988 to 2002: a data-linkage study. Int J Epidemiol. 2006;36(2):310-6.
27. Norwegian Centre for Addiction Research. The Norwegian Prison Release Study - nPRIS 2023. Available from: https://en.prisud.no/npris (accessed 12 January 2023).
28. Ministry of Transport. *Lov om vegtrafikk (LOV-1965-06-18-4) [Road traffic act]*. Oslo, Norway: Ministry of Transport; 2012. Available from Lov om vegtrafikk (vegtrafikkloven) - Lovdata (accessed 12 January 2023)
29. Vindenes V, Jordbru D, Knapskog AB, *et al*. Impairment based legislative limits for driving under the influence of non-alcohol drugs in Norway. Forensic Sci Int. 2012;219(1-3):1-11.
30. Ministry of Transport. *Forskrift om påvirkning av andre rusmidler enn alkohol (FOR-2012-01-20-85) [Regulations on fixed limits for impairment by other intoxicating or anaesthetic/sedative substances than alcohol etc.]*. Oslo, Norway: Ministry of Transport; 2022. Available from Forskrift om faste grenser for påvirkning av andre berusende eller bedøvende middel enn alkohol m.m - Lovdata (accessed 12 January 2023)
31. Fair H, Walmsley R. World Prison Population List. Institute for Crime & Justice Policy Research; 2021. Available from: world_prison_population_list_13th_edition.pdf (prisonstudies.org) (accessed 11 May 2022)
32. Bukten A, Stavseth MR. Suicide in prison and after release: a 17-year national cohort study. Eur J of Epidemiol. 2021;36(10):1075-83.
33. The Norwegian Correctional Services. Annual Statistics 2020. Lillestrøm; 2021. Available from: Årstatistikk+2020+-+Endelig+KDI.pdf (accessed 6 April 2022)
34. The Norwegian Correctional Services. 2020 Annual Report. Lillestrøm; 2021. Available from: arsrapport_kdi_2020.pdf (regjeringen.no) (accessed 6 April 2022)
35. Pedersen AG, Ellingsen CL. Data quality in the Causes of Death Registry. Tidsskrift Nor Laegeforen. 2015;135(8):768-70.
36. The Directorate of e-health. The ICD-10 (and ICD-11) coding system. Available from: https://www.ehelse.no/kodeverk-terminologi/kodeverket-icd-10-og-icd-11 (accessed 23 September 2022)
37. Statistics Norway. Offences and victims reported to the police 2022. Available from: https://www.ssb.no/en/sosiale-forhold-og-kriminalitet/kriminalitet-og-rettsvesen/statistikk/anmeldte-lovbrudd-og-ofre (accessed 11 January 2023)
38. Fazel S, Seewald K. Severe mental illness in 33,588 prisoners worldwide: systematic review and meta-regression analysis. Br J Psychiatry. 2012;200(5):364-73.
39. Fazel S, Yoon IA, Hayes AJ. Substance use disorders in prisoners: an updated systematic review and meta-regression analysis in recently incarcerated men and women. Addiction. 2017;112(10):1725-39.

For peer review only - http://bmjopen.bmj.com/site/about/guidelines.xhtml

40. Karjalainen K, Lintonen T, Impinen A, *et al*. Mortality and causes of death among drugged drivers. J Epidemiology Community Health. 2010;64(6):506-12.
41. Zuckerman M. Sensation Seeking: Behavioral Expressions and Biosocial Bases. In: Wright JD, editor. International Encyclopedia of the Social & Behavioral Sciences (Second Edition). Oxford: Elsevier; 2015. p. 607-14.
42. Sarramon C, Verdoux H, Schmitt L, *et al*. Addiction and personality traits: sensation seeking, anhedonia, impulsivity. Encephale. 1999;25(6):569-75.
43. Roth M, Schumacher J, Brähler E. Sensation seeking in the community: Sex, age and sociodemographic comparisons on a representative German population sample. Pers Individ Dif. 2005;39(7):1261-71.
44. Nordentoft M, Wahlbeck K, Hällgren J, *et al*. Excess mortality, causes of death and life expectancy in 270,770 patients with recent onset of mental disorders in Denmark, Finland and Sweden. PloS One. 2013;8(1):e55176.
45. Skurtveit S, Christophersen AS, Beylich KM, *et al*. Study of rearrests for drunken driving in Norway. Forensic Sci Int. 1998;92(1):21-8.
46. Impinen A, Rahkonen O, Karjalainen K, *et al*. Substance use as a predictor of driving under the influence (DUI) rearrests. A 15-year retrospective study. Traffic Inj Prev. 2009;10(3):220-6.
47. LaBrie RA, Kidman RC, Albanese M, *et al*. Criminality and continued DUI offense: criminal typologies and recidivism among repeat offenders. Behav Sci Law. 2007;25(4):603-14.
48. Bukten A, Herskedal A, Skurtveit S, *et al*. Driving under the influence (DUI) among patients in opioid maintenance treatment (OMT): a registry-based national cohort study. Addiction. 2013;108(11):1954-61.
49. Barta WD, Fisher V, Hynes P. Decreased re-conviction rates of DUI offenders with intensive supervision and home confinement. Am J Drug Alcohol Abuse. 2017;43(6):742-6.
50. Miller PG, Curtis A, Sønderlund A, *et al*. Effectiveness of interventions for convicted DUI offenders in reducing recidivism: a systematic review of the peer-reviewed scientific literature. Am J Drug Alcohol Abuse. 2015;41(1):16-29.
51. World Health Organization. Status report on prison health in the WHO European Region. Copenhagen: WHO Regional Office for Europe; 2019.

For peer review only

For peer review only - http://bmjopen.bmj.com/site/about/guidelines.xhtml

Supplementary tables

Supplementary table 1. Mean age at death (with standard deviation) for all causes of death

Cause of death	No DUI	DUI		
		DUI only	DUI drug	DUI other
Cancer	58.87 (10.93)	62.97 (9.29)	58.90 (10.64)	61.87 (9.23)
Circulatory	56.70 (12.55)	60.80 (10.77)	57.02 (12.24)	59.18 (10.63)
Respiratory	58.99 (11.33)	63.55 (10.43)	60.67 (13.55)	63.50 (12.72)
Digestive	57.08 (10.82)	56.82 (7.59)	54.00 (11.90)	54.00 (8.41)
Alcoholic liver disease	52.12 (7.91)	56.53 (7.24)	54.46(7.92)	52.31 (5.49)
Other internal	51.47 (14.18)	60.25 (11.53)	51.76 (13.74)	60.90 (15.27)
**Total internal causes**	**56.57 (12.23)**	**61.12 (10.20)**	**56.95 (12.13)**	**59.57 (10.93)**
Transport-related	36.62 (11.40)	49.54 (7.98)	38.33 (12.09)	38.47 (12.38)
Intoxication[1]	38.45 (11.38)	48.49 (13.23)	40.27 (12.02)	46.21 (13.23)
Alcohol-related	54.08 (9.53)	57.89 (9.39)	54.96 (11.81)	54.61 (10.25)
Drug-related	36.59 (10.08)	40.56 (10.54)	37.44 (9.80)	39.54 (11.48)
Suicide	37.51 (11.30)	42.78 (14.65)	37.37 (9.75)	41.32 (12.89)
Homicide	36.19 (11.06)	-	41.00 (9.68)	-
Other accident-related	45.14 (14.84)	50.62 (11.91)	47.08 (14.88)	49.97 (15.60)
**Total external causes**	**38.69 (11.93)**	**47.75 (13.32)**	**40.38 (12.22)**	**45.11 (13.88)**
Unexplained	50.94 (13.25)	58.34 (11.99)	53.90 (12.81)	53.00 (12.02)
Unknown cause	47.21 (13.32)	55.72 (12.99)	48.52 (13.55)	54.73 (10.81)
**Total deceased**	**46.42 (14.86)**	**56.39 (13.03)**	**47.62 (14.63)**	**52.77 (14.12)**

[1] Alcohol- and drug-related combined

For peer review only

STROBE Statement—Checklist of items that should be included in reports of ***cohort studies***

	Item No	Recommendation	Page No
**Title and abstract**	1	(*a*) Indicate the study's design with a commonly used term in the title or the abstract<br>(*b*) Provide in the abstract an informative and balanced summary of what was done and what was found	1-3
**Introduction**			
Background/rationale	2	Explain the scientific background and rationale for the investigation being reported	4-5
Objectives	3	State specific objectives, including any prespecified hypotheses	5
**Methods**			
Study design	4	Present key elements of study design early in the paper	6
Setting	5	Describe the setting, locations, and relevant dates, including periods of recruitment, exposure, follow-up, and data collection	6
Participants	6	(*a*) Give the eligibility criteria, and the sources and methods of selection of participants. Describe methods of follow-up<br>(*b*) For matched studies, give matching criteria and number of exposed and unexposed	6-7
Variables	7	Clearly define all outcomes, exposures, predictors, potential confounders, and effect modifiers. Give diagnostic criteria, if applicable	7-8
Data sources/ measurement	8*	For each variable of interest, give sources of data and details of methods of assessment (measurement). Describe comparability of assessment methods if there is more than one group	6-7
Bias	9	Describe any efforts to address potential sources of bias	8
Study size	10	Explain how the study size was arrived at	6
Quantitative variables	11	Explain how quantitative variables were handled in the analyses. If applicable, describe which groupings were chosen and why	8-9
Statistical methods	12	(*a*) Describe all statistical methods, including those used to control for confounding<br>(*b*) Describe any methods used to examine subgroups and interactions<br>(*c*) Explain how missing data were addressed<br>(*d*) If applicable, explain how loss to follow-up was addressed<br>(*e*) Describe any sensitivity analyses	8-9
**Results**			
Participants	13*	(a) Report numbers of individuals at each stage of study—eg numbers potentially eligible, examined for eligibility, confirmed eligible, included in the study, completing follow-up, and analysed<br>(b) Give reasons for non-participation at each stage<br>(c) Consider use of a flow diagram	6,9
Descriptive data	14*	(a) Give characteristics of study participants (eg demographic, clinical, social) and information on exposures and potential confounders<br>(b) Indicate number of participants with missing data for each variable of interest<br>(c) Summarise follow-up time (eg, average and total amount)	10
Outcome data	15*	Report numbers of outcome events or summary measures over time	10-11

For peer review only

Main results	16	(*a*) Give unadjusted estimates and, if applicable, confounder-adjusted estimates and their precision (eg, 95% confidence interval). Make clear which confounders were adjusted for and why they were included<br>(*b*) Report category boundaries when continuous variables were categorized<br>(*c*) If relevant, consider translating estimates of relative risk into absolute risk for a meaningful time period	12-13
Other analyses	17	Report other analyses done—eg analyses of subgroups and interactions, and sensitivity analyses	-
**Discussion**			
Key results	18	Summarise key results with reference to study objectives	14
Limitations	19	Discuss limitations of the study, taking into account sources of potential bias or imprecision. Discuss both direction and magnitude of any potential bias	16
Interpretation	20	Give a cautious overall interpretation of results considering objectives, limitations, multiplicity of analyses, results from similar studies, and other relevant evidence	14-16
Generalisability	21	Discuss the generalisability (external validity) of the study results	16
**Other information**			
Funding	22	Give the source of funding and the role of the funders for the present study and, if applicable, for the original study on which the present article is based	18

*Give information separately for exposed and unexposed groups.

Note: An Explanation and Elaboration article discusses each checklist item and gives methodological background and published examples of transparent reporting. The STROBE checklist is best used in conjunction with this article (freely available on the Web sites of PLoS Medicine at http://www.plosmedicine.org/, Annals of Internal Medicine at http://www.annals.org/, and Epidemiology at http://www.epidem.com/). Information on the STROBE Initiative is available at http://www.strobe-statement.org.

For peer review only

BMJ Open

All-cause and cause-specific mortality among individuals imprisoned for Driving under the Influence of alcohol and drugs in Norway (2000-2016): a retrospective cohort study

Journal:	*BMJ Open*
Manuscript ID	bmjopen-2023-078848.R1
Article Type:	Original research
Date Submitted by the Author:	23-Nov-2023

Complete List of Authors:	Jamt, Ragnhild; Oslo University Hospital, Department of Forensic Sciences<br>Bukten, Anne; University of Oslo, Norwegian Centre For Addiction Research; Oslo University Hospital, Section for Clinical Addiction Research<br>Stavseth, Marianne ; Oslo University Hospital, Section for Clinical Addiction Research; University of Oslo, Norwegian Centre for Addiction Research<br>Bogstrand, Stig Tore; Oslo University Hospital, Department of Forensic Sciences; University of Oslo, Department of Public Health Science<br>Tverborgvik, Torill; University of Oslo, Norwegian Centre for Addiction Research

**Primary Subject Heading**:	Epidemiology
Secondary Subject Heading:	Epidemiology
Keywords:	REGISTRIES, EPIDEMIOLOGY, Retrospective Studies

SCHOLARONE™ Manuscripts

BMJ

I, the Submitting Author has the right to grant and does grant on behalf of all authors of the Work (as defined in the below author licence), an exclusive licence and/or a non-exclusive licence for contributions from authors who are: i) UK Crown employees; ii) where BMJ has agreed a CC-BY licence shall apply, and/or iii) in accordance with the terms applicable for US Federal Government officers or employees acting as part of their official duties; on a worldwide, perpetual, irrevocable, royalty-free basis to BMJ Publishing Group Ltd ("BMJ") its licensees and where the relevant Journal is co-owned by BMJ to the co-owners of the Journal, to publish the Work in this journal and any other BMJ products and to exploit all rights, as set out in our licence.

The Submitting Author accepts and understands that any supply made under these terms is made by BMJ to the Submitting Author unless you are acting as an employee on behalf of your employer or a postgraduate student of an affiliated institution which is paying any applicable article publishing charge ("APC") for Open Access articles. Where the Submitting Author wishes to make the Work available on an Open Access basis (and intends to pay the relevant APC), the terms of reuse of such Open Access shall be governed by a Creative Commons licence – details of these licences and which Creative Commons licence will apply to this Work are set out in our licence referred to above.

Other than as permitted in any relevant BMJ Author's Self Archiving Policies, I confirm this Work has not been accepted for publication elsewhere, is not being considered for publication elsewhere and does not duplicate material already published. I confirm all authors consent to publication of this Work and authorise the granting of this licence.

For peer review only

All-cause and cause-specific mortality among individuals imprisoned for Driving under the Influence of alcohol and drugs in Norway (2000-2016): a retrospective cohort study

Ragnhild E. G. Jamt[1], Anne Bukten[2,3,4], Marianne Riksheim Stavseth[2,3], Stig T. Bogstrand[1,5], Torill Tverborgvik[2]

[1]Department of Forensic Sciences, Oslo University Hospital, Oslo, Norway

[2]Norwegian Centre for Addiction Research, University of Oslo, Oslo, Norway

[3]Section for Clinical Addiction Research, Oslo University Hospital, Oslo, Norway

[4]University College of Norwegian Correctional Service, Lillestrøm, Norway

[5]Department of Public Health Science, University of Oslo, Oslo, Norway

Corresponding author:

Ragnhild E. G. Jamt

Postal address: Oslo University Hospital, Department of Forensic Sciences,

Post box 4950 Nydalen, 0424 Oslo, Norway

E-mail address: rmraej@ous-hf.no

For peer review only

Abstract

Aims:

To describe all-cause and cause-specific mortality and to investigate factors associated with mortality among individuals imprisoned for driving under the influence (DUI) of alcohol and psychoactive drugs in the Norwegian prison population.

Design:

Retrospective cohort study. The Norwegian prison registry was linked to the Norwegian Cause of Death Registry (2000-2016).

Setting:

Norway

Participants/Cases:

The cohort consisted of 96 856 individuals imprisoned in Norway over a 17-year period obtained from the Norwegian prison registry.

Primary and secondary outcome measures:

Adjusted odds ratios (aOR) with 95% confidence intervals (CI) were calculated for death due to any, natural and unnatural causes of death. Analyses were stratified according to DUI convictions: no DUI convictions, only DUI convictions (DUI only), DUI and at least one other drug and alcohol conviction (DUI drug), and DUI and at least one conviction other than drug and alcohol conviction (DUI other).

Results:

In total, 29.3% individuals had one or more imprisonments for DUI The risk of all-cause mortality was elevated for those convicted for DUI, but only in combination with other types of crimes (DUI drug: aOR = 1.5, 95% CI = 1.4-1.6, DUI other: aOR = 1.2, 95% CI = 1.1-1.4) . The risk of death from natural causes was significantly elevated for all individuals convicted for DUI (DUI only: aOR = 1.9, 95% CI = 1.7-2.1, DUI drug: aOR: 1.6, 95% CI = 1.5-1.8, DUI other: aOR = 1.7, 95% CI = 1.5-2.0). The risk of death from unnatural causes was lower for DUI only (aOR = 0.8, 95CI = 0.7-0.9) and elevated for DUI drug (aOR = 1.6, 95% CI = 1.5-1.7).

For peer review only - http://bmjopen.bmj.com/site/about/guidelines.xhtml

Conclusions:

The risk of all-cause mortality was significantly elevated for those convicted of DUI, but only in combination with other types of crimes.

Strengths and limitations:

- We were able to study the mortality of the entire Norwegian prison population through linkage of mandatory national registries during an observation period of 17 years.
- Linkage of data through unique Personal Identifiqation Number (PINs) reduces the chances of linkage bias and loss to follow up.
- The lack of information on background socio-demographics and health variables in the dataset implies that we could not adjust for important pre-existing conditions associated with mortality.
- Data on individuals who served conditional sentences outside prison for DUI was not available.

For peer review only

For peer review only - http://bmjopen.bmj.com/site/about/guidelines.xhtml

INTRODUCTION

The literature on driving under the influence (DUI) of psychoactive substances (alcohol, psychoactive medicinal drugs, and illicit drugs) has mainly revolved around topics such as the prevalence of DUI and the risks associated with road traffic injuries and deaths (1-5). DUI poses a threat to the lives and safety of people who engage in DUI (6), as well as other road users (7); however, the research regarding future outcomes of people convicted of driving under the influence of alcohol and drugs is both limited and somewhat outdated (8, 9).

Drivers who engage in DUI have been characterized by serious health problems such as substance use and dependence, major depressive disorders, anxiety disorders, hyperactivity disorders and risk-taking dispositions (8). People arrested for DUI exhibit similar patterns of health problems and mortality as found among people with alcohol use disorders (10). Studies by Gjerde et al. and Ruud et al. found that between 35-60% of those arrested for DUI in Norway had an alcohol use disorder (11-13). In Germany, more than 80% of persons arrested for DUI of alcohol had a blood alcohol concentration (BAC) higher than 1.9 g/dl and could be characterized as having alcohol use disorders (14). People convicted of DUI have also been characterized by lower levels of education and higher rates of unemployment, disability pensions and divorce compared to the general population (15).

Norway has practiced a per se law for driving under the influence of alcohol since 1936. Since 2001, the legal limit in Norway for DUI of alcohol in blood is 0.02 g/dl. Graded sanctioning is practiced; a driver is sentenced to conditioned imprisonment for driving with BACs between 0.05g/dl and 0.12 g/dl, and unconditioned imprisonment for BACs above 0.12 g/dl (16). *Per se* limits for 28 psychoactive drugs were implemented in 2012/2016 corresponding to a BAC of 0.02 g/dl (17). These psychoactive drugs includes both medicinal and illicit drugs such as benzodiazepines, cannabis, opioids and psychostimulants (amphetamines, cocaine). In addition, limits for graded sanction corresponding to BACs of 0.05 and 1.2 g/dl for 24 of these drugs have been implemented (18).

The most recent roadside survey in Norway was performed in 2016/2017 and included 5034 drivers (19). The total prevalence of alcohol and psychoactive drugs among random drivers in this study was around 5%. While the prevalence of drivers with a BAC above the legal limit of 0.02 g/dl was 0.2%, the total prevalence of psychoactive medicinal drugs and of illicit drugs were 3.0% and 1.7%,

For peer review only - http://bmjopen.bmj.com/site/about/guidelines.xhtml

respectively. The prevalence of drugs in concentrations above the legislative limits was 1.1% for medicinal drugs and 0.7% for illicit drugs. Hence, driving under the influence of alcohol is less common in Norway. Legislation on DUI, including strong law enforcement and strict punishments over many years is likely to have contributed to the low numbers of DUI in Norway (20). However, the prevalence of psychoactive substances among drivers arrested on suspicion of DUI and among drivers involved in road traffic crashes is high (21, 22). A paper reviewing the risk of serious road traffic crashes while engaging in DUI found higher risks in Norwegian and Finnish studies than in studies from other countries (23). It has been suggested that in countries with a strong law enforcement and strict punishments for DUI, people driving under the influence are a more selected group with higher incidence of substance use disorders (SUDs) along with higher levels of sensation-seeking and risk-taking behavior (21). It is furthermore suggested that people engaging in risky driving behavior might be less affected by threats of punishment (24).

Norway is characterized by having low rates of imprisonment. In 2021, the prison population rate per 100 000 of the national population was 56 in Norway, compared to 629 in the US and 131 in the UK (25). The Norwegian penal system aim at rehabilitation in that the sentence is recognized as the punishment, and universal health care, including drug treatment, is to be provided in prison as it is outside of prison (26).

Norwegian prison units are public and are located all over Norway. This prison organization allows most prisoners to preserve geographical closeness to friends and family. In 2020, 18% of all unconditional sentences lasted less than 30 days and 76% lasted up to one year (27). Women constitute a minority in Norwegian prisons, with an annual proportion of approximately 5% (28).

In 2019, 21% of all prison sentences in Norway included at least one DUI-offence (29). Several studies have demonstrated that the prison population has a greater risk of premature death compared to the general population, particularly in the short-term period following release from prison (30-33). A study based on the Norwegian police registration system showed that the risk of premature death is particularly high among individuals arrested for alcohol and drug related crimes, compared to both the general population and individuals arrested for other criminal offences (9).

Although there are similarities between people convicted of DUI and individuals with alcohol use disorders in terms of health problems, the entire group of people convicted of DUI is large and

For peer review only - http://bmjopen.bmj.com/site/about/guidelines.xhtml

heterogeneous, and therefore more specific knowledge on the risk factors and mortality is warranted for this group (10).

Using data on all individuals imprisoned in Norway in the period 2000-2016, we defined three sub-categories of the DUI group in order to examine combinations of DUI convictions and other convictions in relation to mortality. The aims of this paper were to:

1. describe the different groups of individuals imprisoned for DUI in the Norwegian prison population

2. describe all-cause and cause-specific mortality in the DUI-groups and compare with individuals imprisoned for other crimes than DUI, and

3. investigate factors associated with natural and unnatural mortality in the prison population.

METHODS

Design, study population and data sources

In this retrospective cohort study, we used data from the Norwegian Prison Release Study (nPRIS) (34), including all persons imprisoned in Norway over a 17-year period (January 1st 2000, until December 31st 2016). The nPRIS-cohort constitutes 114 745 individuals collected from the Norwegian Prison Registry. The registry serves administrative and statistical purposes and includes personal data on all persons imprisoned in Norway, including age, sex, convictions, and sentences (26). The nPRIS-cohort includes individuals who started serving a prison sentence, had an ongoing prison sentences and individuals who were released from prison during the observation period. Information on any prison sentences prior to the start of the observation period started were not available.

Data from the nPRIS-cohort were linked to the Norwegian Cause of Death Register (NCoDR) through the unique 11-digit Norwegian Personal Identification Number (PIN). The PIN isgiven to all persons who are born in Norway, have a valid residence permit for at least six months or have officially moved to Norway. The Norwegian Cause of Death Registry (NCoDR) includes death certificates reported by medical doctors after examination of the deceased. The NCoDR includes information on time of death along with the underlying cause of death (the disease or injury which

For peer review only - http://bmjopen.bmj.com/site/about/guidelines.xhtml

initiated the chain of morbid events leading directly to death) and the immediate causes of death (the terminal event or complication present at the time of death) (35). All causes of death are coded using the International Classification of Diseases, 10th revision (ICD-10) (36).

In total, 19 140 individuals were excluded from the study cohort due to lack of a valid PIN. Hence, the study population consisted of 96 856 individuals, including both those who died and those who lived during the observation period, contributing to 167 068 releases.

For peer review only

Measures

Individuals in the cohort who had one or more convictions for DUI were devided into three subgroups: *DUI-only* as those having no other convictions but DUIs during the observation period; *DUI-drug* as those having both DUI and at least one other drug and alcohol conviction during the observation period; and *DUI-other* as those having DUI and at least one other conviction other than drug and alcohol conviction during the observation period.

We categorized all causes of death as either natural or unnatural, as described in Table 1. Natural causes were defined as ICD-10 chapters A through Q, while unnatural causes were defined as ICD-10 chapters V through Y. Drug- and alcohol-related causes (F10-F19) were defined as unnatural causes of death (Table 1).

We classified types of convictions according to the nine-group classification used for Statistics Norway's official crime statistics (37). In their statistics, DUI offences are included in the category "Drug and alcohol offences", but as we wanted to study this offence in particular, we constructed a subgroup with DUI convictions exclusively. Thus, the ten-group classification used in this paper were: Property theft; Other offences for profit; Criminal damage; Drug and alcohol offences (not including DUI); Public order and integrity violations; Sexual offences; Traffic offences; Violence and maltreatment; Other offences; and DUI. One imprisonment can include multiple convictions. Convictions were aggregated across the study period, and dummy coded as "convicted of" (1) or "not convicted of" (0).

For peer review only - http://bmjopen.bmj.com/site/about/guidelines.xhtml

Table 1. Categorization of causes of death according to ICD-10 codes.

**Cause of death**	**ICD-10 code**
Cancer	C00-C97
Circulatory	I00-I99
Respiratory	J00-J99
Digestive	K00-K69, K71-K93
Alcoholic liver disease	K70
Other natural	A,B,D-H,L-Q
Natural causes of death	All codes listed above
Transport-related	V, Y85
Intoxication[1]	
Alcohol-related	F10,X45,Y15
Drug-related	F11,F12,F14-16,F19, X41,X42,X44, Y11,Y12,Y14
Suicide	X60-X84
Homicide	X85-X99, Y00-Y09
Other accident-related	W,X2,Y2
Unnatural causes of death	All codes listed above (excluding natural causes)
Unexplained	R95-R99
Unknown cause	No ICD-10 code recorded in NDR

[1] Alcohol- and drug-related causes combined

Statistical analyses

All analyses were performed using SPSS, version 26. We made group comparisons using Student's t-test or Mann-Whitney U-test for continuous data and X^2-test for categorical data. Kruskal-Wallis test was used for testing distribution between sub-groups.

The associations between mortality and DUI convictions were examined using logistic regression. The coefficients were interpreted as odds ratios (OR) with 95% confidence intervals (95% CI). Individuals who died due to natural causes differed significantly from those who died due to unnatural causes. Therefore, separate logistic regression models were fitted. The three regression models were defined with the following outcome of interest 1) all-cause mortality (n=8053) 2) death due to natural causes (n=3379), and 3) death due to unnatural causes (n=4139). To avoid competing risks, unknown and unnatural cause deaths were excluded from analysis in the second regression model, and unknown and natural cause deaths were excluded from analysis in the third model. Univariate regression models were fitted, and statistically significant covariates (p-value < 0.05) were included in the multivariable analysis.

For peer review only - http://bmjopen.bmj.com/site/about/guidelines.xhtml

Patient and public involvement

Patients and/or the public were not involved in the design, or conduct, or reporting, or dissemination plans of this research.

RESULTS

Participants

The cohort consisted of 96 856 individuals of which 28 393 individuals (29.3%) were convicted of any DUI (Table 2). Among these, 8898 (9.2%) were only convicted of DUI (DUI-only), 16 159 (16.7%) were convicted of DUI and at least one drug and alcohol offence (DUI-drug), and 3336 (3.4%) were convicted of DUI and at least one crime other than drug or alcohol related offences (DUI-other) during the observation period (Table 2). The DUI-only group had the highest proportion of women (13.7%) and were the oldest at last incarceration (mean age 38.4 years). There were significant differences in the mean age at last incarceration between the groups (results not shown).

Approximately one third of the total cohort (n = 30 506) had more than one imprisonment during the observation period. In the DUI-drug group, almost 60% had more than one imprisonment; 21 % had more than one imprisonment for DUI. Only 5.5% of the DUI-only group had multiple incarcerations for DUI during the study period. The overall proportion of deceased among those convicted of any DUI was 10.5% compared to 7.4% among the non-DUI-offenders. The proportion of deceased was highest in the DUI-drug group (11.3%) (Table 2).

For peer review only - http://bmjopen.bmj.com/site/about/guidelines.xhtml

Table 2. Background characteristics of the study population (n=96 856), 1st January 2000 – 31st December 2016.

	No DUI		DUI					
			DUI only		DUI drug		DUI other	
	n	%	n	%	n	%	n	%
Total	68463	70.7	8898	9.2	16159	16.7	3336	3.4
Sex[1]								
Men	61171	89.3	7680	86.3	14870	92.0	3155	94.6
Women	7275	10.6	1218	13.7	1289	8.0	181	5.4
Age at last conviction[2,3]	34.6 (11.8)		38.4 (14.1)		36.2 (11.4)		36.6 (13.4)	
Number of conviction[3,4]	1.6 (1.4)		1.1 (0.3)		2.7 (2.6)		1.6 (0.9)	
Convictions[5]								
Property theft	9974	14.6	-	-	5063	31.3	351	10.5
Other offences for profit	16335	23.9	-	-	4197	26.0	358	10.7
Criminal damage	4045	5.9	-	-	2102	13.0	171	5.1
Drug and alcohol offences[6]	28505	41.6	-	-	16159	100	-	-
Public order/integrity	15379	22.5	-	-	5673	35.1	652	19.5
Sexual offences	5362	7.8	-	-	333	2.1	124	3.7
Traffic offences	3648	5.3	-	-	5372	33.2	1932	57.9
Violence and maltreatment	25597	37.4	-	-	4932	30.8	1061	31.8
Other offences	2278	3.3	-	-	947	5.9	89	2.7
Driving under the influence	-	-	8898	100	16159	100	3336	100
1DUI conviction	-	-	*8657*	*97.3*	*12755*	*78.9*	*3032*	*90.9*
2+DUI convictions	-	-	*241*	*2.7*	*3404*	*21.1*	*304*	*9.1*
Deceased	5064	7.4	825	9.3	1825	11.3	339	10.2

[1] 17 persons missing information on sex
[2] 42 persons missing information on age
[3] Continuous measures presented with mean (SD)
[4] During the study period
[5] During the study period. Note: A person may have several convictions per incarceration and several incarcerations
[6] Not including DUI

For peer review only

Causes of death

Deaths among those convicted of DUI-only were mostly due to natural causes (58.9%; Table 3). Diseases of the circulatory system and cancer were the predominant natural causes of death in all groups. A higher proportion of death due to alcoholic liver disease was found among those convicted of DUI-only (5.2%). Those convicted of DUI-only had the lowest proportion of unnatural causes of death (33.0%).

For peer review only - http://bmjopen.bmj.com/site/about/guidelines.xhtml

Deaths among those convicted of DUI-drug were more often due to unnatural causes (54.1%), as was the case among those convicted of other crimes than DUI (53.9%). Intoxication (drug or alcohol related) accounted for the largest proportion of the unnatural causes of death in all groups. Among those convicted of DUI-drug, the vast majority of the deaths caused by intoxications were drug-related.

About 5% of mortalities in the DUI-drug and DUI-other groups were transport related. The proportion in the DUI-only group was 1.6%.

The DUI-only group was in general older at time of death than the other groups. Mean age at death was 61 years among DUI-only, but only 48 years in the DUI-drug group (Supplementary Table 1). Overall, the non-DUI-offenders had the lowest mean age at death of 46 years.

Table 3. Cause of death (n, %) stratified by DUI-groups.

	No DUI		DUI					
			DUI only		DUI drug		DUI other	
**Cause of death**	n	%	n	%	n	%	n	%
Cancer	640	12.6	144	17.5	214	11.7	46	13.6
Circulatory	620	12.2	158	19.1	234	12.8	62	18.3
Respiratory	215	4.2	55	6.7	86	4.7	12	3.5
Digestive	98	1.9	22	2.7	43	2.4	7	2.1
Alcoholic liver disease	105	2.1	43	5.2	56	3.1	16	4.7
Other natural	325	6.4	64	7.8	94	5.2	20	5.9
**Total** natural **cause**	**2003**	**39.6**	**486**	**58.9**	**727**	**39.8**	**163**	**48.1**
Transport-related	224	4.4	13	1.6	90	4.9	17	5.0
Intoxication[1]	1606	31.8	164	19.9	601	32.9	70	20.6
Alcohol-related	*170*	*3.4*	*75*	*9.1*	*97*	*5.3*	*31*	*9.1*
Drug-related	*1436*	*28.4*	*89*	*10.8*	*504*	*27.6*	*39*	*11.5*
Suicide	569	11.2	54	6.5	160	8.8	28	8.3
Homicide	69	1.4	-	-	31	1.7	-	-
Other accident-related	262	5.2	39	4.7	106	5.8	31	9.1
**Total** unnatural **cause**	**2730**	**53.9**	**272**	**33.0**	**988**	**54.1**	**149**	**44.0**
Unexplained	140	2.8	35	4.2	52	2.8	12	3.5
Unknown cause	191	3.8	32	3.9	58	3.2	15	4.4
**Total deceased**	**5064**	**100**	**825**	**100**	**1825**	**100**	**339**	**100**

[1] Alcohol- and drug-related combined
- Cells with n < 5 are not displayed

For peer review only - http://bmjopen.bmj.com/site/about/guidelines.xhtml

Factors associated with mortality

After adjusting for relevant covariates, being male and increasing age at last incarceration were factors significantly associated with elevated risk of death from all causes: Sex: (aOR: 1.2, CI: 1.1-1.3); Age: (aOR: 1.1, CI: 1.1-1.1). This was also the case for natural causes: Sex: (aOR: 1.2, CI: 1.0-1.3); Age: (aOR: 1.1, CI: 1.1-1.1), and unnatural causes: Sex: (aOR: 1.1, CI: 1.0-1.2); Age: (aOR: 1.0, CI: 1.0-1.0) (Table 4). Number of convictions was not significantly associated with death from all causes (aOR: 0.99, CI: 0.97-1.00). However, the variable was a significant protective factor on death from natural causes (aOR: 0.9, CI: 0.8-0.9) and was a significant risk factor on death from unnatural causes (aOR: 1.1, CI:1.0-1.1).

Being convicted of DUI and other crimes significantly elevates the risk of death from all causes (DUI-drug: aOR 1.5, CI: 1.4-1.6, DUI-other: aOR 1.2, CI: 1.1-1.4). The same was true for death from natural causes (DUI-drug: aOR 1.8, CI: 1.6-2.0; DUI-other: aOR 1.3, CI: 1.1-1.6). While being convicted of DUI and one or more drug and alcohol related crimes increased the risk of dying from unnatural causes (aOR 1.5, CI: 1.3-1.6), being convicted of DUI only had a significant protective effect on the risk of dying from unnatural causes (aOR 0.8, CI: 0.7-0.9).

For peer review only - http://bmjopen.bmj.com/site/about/guidelines.xhtml

Table 4 Summary of the logistic regression models for all-cause, natural and unnatural causes of death (for full table see Supplemantary table 2). Estimates are given as adjusted odds ratios (aOR) with corresponding 95% confidence intervals. The level of statistical significans was $p \leq 0.05$

	**All cause of death, n=8053**		**Natural causes of death, n=3379**		**Unatural causes of death, n=4139**	
	aOR[1] (95% CI)	***p***	aOR[1] (95% CI)	***p***	aOR[1] (95% CI)	***p***
Sex (female ref.)	1.22 (1.12-1.32)	**<0.001**	1.18 (1.04-1.33)	**0.011**	1.11 (1.00-1.24)	**0.063**
Age[2]	1.06 (1.05-1.06)	**<0.001**	1.11 (1.10-1.11)	**<.001**	1.01 (1.01-1.02)	**<.001**
No. of convictions	0.99 (0.97-1.00)	**0.092**	0.85 (0.83-0.88)	**<0.001**	1.06 (1.04-1.08)	**<0.001**
Conviction (no dui ref.):	-		-		-	
DUI-only	0.99 (0.91-1.07)	**0.916**	1.11 (0.99-1.24)	**0.066**	0.78 (0.69-0.89)	**<.001**
DUI-drug	1.51 (1.44-1.63)	**<.001**	1.80 (1.64-1.98)	**<.001**	1.45 (1.34-1.57)	**<.001**
DUI-other	1.23 (1.09-1.38)	**<.001**	1.34 (1.12-1.60)	**0.001**	1.13 (0.95-1.34)	**0.164**

[1]Adjusted for covariates significant in crude analyses.
[2]Age at last incarceratinon

For peer review only - http://bmjopen.bmj.com/site/about/guidelines.xhtml

For peer review only

DISCUSSION

In this retrospective cohort study of mortality among people imprisoned for DUI in Norway, we found the risk of all-cause mortality to be significantly elevated for those convicted of DUI, but only in combination with other types of crimes, and especially in combination with other drug and alcohol offences. Being convicted of no other crime than DUI had a protective effect on unnatural cause of death after adjusting for age, sex and number of previous convictions.

Psychiatric disorders and SUDs are highly prevalent in the prison population (38, 39), and a large proportion of people imprisoned for DUI are likely to have some degree of SUD and/or mental health problems (8). In our study, almost half of the total cohort had one or more drug and alcohol convictions and 30% had at least one DUI conviction. Among those convicted of DUI, 60% had additional convictions for other drug and alcohol offences.

Adjusted for several other factors, individuals with a history of both DUI and other drug and alcohol convictions had the highest risk of mortality due to both natural and unnatural causes. The most common unnatural causes were intoxication and suicide and the most common natural causes were diseases of the circulatory system and cancer.This is in line with previous research findings on the most dominant causes of death among people convicted of DUI (10, 40). The high mortality from natural causes might indicate unhealthier lifestyles in aspects other than just alcohol and drug use, like smoking, physical inactivity and obesity (41, 42).

Many DUI-offenders also have a history of convictions for other types of crime in addition to the DUI-offence, and the tendency to relapse into DUI is well known (43-45). Previous research has identified some characteristics of individuals who relapse into DUI. In American, Finish and Swedish studies, DUI relapse has been associated with factors such as being male, young age, mental health disorders, high concentrations of alcohol and drugs at the time of the arrest, and previous DUI convictions (44, 46-48). Particularly those driving under the influence of drugs had high rates of rearrests (44, 48). The results of the present study revealed that those convicted of both DUI and other drug and alcohol offences had on average the highest number of incarcerations during the observation period (2.7 incarcerations)and the largest proportion of individuals withtwo or more convictions for DUI (21.1%) compared to the two other DUI-groups. The proportion of younger males was higher in the DUI-drug group compared to the DUI-only group and about the same as in the DUI-other group.

For peer review only - http://bmjopen.bmj.com/site/about/guidelines.xhtml

Impinen et al., suggests that an over-representation of unnatural causes of death might be an indicator of a risk-taking lifestyle (10). Sensation-seeking behavior is a personality trait that has been associated with engaging in unusual and risky acts, fast and reckless driving, and alcohol and drug use (49, 50). Sensation-seeking behavior has also been shown to decline with age and is usually more pronounced among males (51).

The mean age at death was low for the whole cohort and particularly low for death due to unnatural causes. As expected, higher age was significantly associated with increased risk of death. However, when stratifying on cause of death (natural and unnatural causes), the effect of increasing age at last incarceration became more pronounced for the risk of death from natural causes. In a study by Nordentoft et al. (52) high excess mortality due to somatic illnesses was found among patients with psychiatric disorders and SUDs. The excess mortality was highest among patients with SUDs who died 20 years earlier than the general population.

It is evident from the present study that people convicted of DUI are a heterogenic group with those only imprisoned for DUI standing out from both the other DUI groups and from those imprisoned for other crimes than DUI. We found that the "DUI only"-group were older, had fewer incarcerations, higher proportion of women, and were at significantly lower risk of death from unnatural causes. Previous research have identified socioeconomic status, civil status, pre-exciting health conditions, employment or education and living conditions to significantly predict the mortality in the prison population (53, 54). The fact that the DUI-only group were at lower risk of death from unnatural causes compared to all other studied groups in the present study could for instance be related to factors like higher socioeconomic status and a lower prevalence of pre-exciting drug dependence in this group. Another point to make notice of is the selection of the DUI-only group in this study which is based solely on DUI convictions. This indicates that the individuals of the DUI-only group commits less crime and thereby have fewer incarcerations, which in turn is protective on health and mortality (55).The prison context may offer an important opportunity for interventions in terms of SUD treatment. A Norwegian study on DUI among patients in opioid agonist treatment (OAT) found a 40% reduction in DUI convictions while in treatment. However, patients with two or more convictions for DUI before entering treatment had higher odds for further convictions of DUI during OAT compared with patients having no DUI

For peer review only - http://bmjopen.bmj.com/site/about/guidelines.xhtml

convictions during the pre-treatment period (56). Multiple DUI convictions might thus be viewed as a proxy for the severity of their substance use.

Attempting to combat DUI recidivism and to lower the social and economic costs of incarceration various DUI prevention programs have been developed internationally. An example is the *Intensive supervision programs* (ISPs) (57). These programs include home confinement with activities such as screening and assessment of offenders' substance abuse problems, treatment and education related to DUI behavior, electronic monitoring of offender movements and use of ignition interlock devices or other restrictions on driving. At present, the assessment of the effectiveness of DUI prevention programs is inconclusive. However, there is some evidence to support the effectiveness of programs utilizing intensive supervision and education (58). In Norway, the program consists of education, individual- and group therapy sessions and investigations of the offenders' treatment needs, but is only available for individuals serving conditional sentences, and thus disqualifying people with more severe SUDs typically serving unconditional sentences.

The results of the present study should be interpreted with certain limitations in mind. The lack of information on background socio-demographics and health variables in the dataset implies that we could not adjust for important pre-existing conditions and factors associated with mortality. It is likely that such factors also would have affected the findings in the present study if the information had been available. Data on individuals who served conditional sentences and served sentences with electronic monitoring was not available for the present study. Thus, our data contains a selection bias as we only have data on the most severe cases of legal offences who likely have a greater disease burden and higher mortality. A strength of this study is that we were able to study the mortality of the entire Norwegian prison population through linkage of mandatory national registries during an observation period of 17 years. The linkage of datasets through unique PINs reduces the chances of linkage bias and loss to follow up is rare. Furthermore, the classification of all deaths is in accordance with the ICD-10 criteria and cause of death are reported by a clinician according to individual ICD codes, reducing the chances of information bias. The NCoDR is a reliable source of data on causes of death with combined degree of coverage and completeness of 98% of the deaths in Norway (35).

Systematic screening with respect drug and alcohol use disorders is a critically important first step to identify individuals who should be offered treatment during incarceration. However, validated

For peer review only - http://bmjopen.bmj.com/site/about/guidelines.xhtml

screening tools are scarcely implemented in European prisons (59). The results from our study imply that in places where systematic screening is not routinely carried out data on current and prior offence(s) a person is sentenced to prison for (e.g., DUI, other alcohol and drug offences) might serve as an indicator of harmful alcohol and drug use. This data is readily available to the correctional service staff, and can be used as means for further investigations using validated screening tools to properly identify people with potential harmful alcohol and drug use. Moreover, when risk has been identified, provision of evidence-based treatment and harm reduction services like intensive supervision and education programs must be provided for those in need.

For peer review only

For peer review only - http://bmjopen.bmj.com/site/about/guidelines.xhtml

Acknowledgments: We thank the Directorate of the Norwegian Correctional Services and the Norwegian Cause of Death Registry for valuable assistance regarding registry data.

Competing interests: none declared.

Funding: This work was supported by the South-Eastern Norway Regional Health Authority: grant number: 2019091 and the Norwegian Research Council: grant number: 301535.

Contributors: R. E. G. Jamt: Conceptualization, Formal analysis, Investigation, Methodology, Writing – original draft A. Bukten: Conceptualization, Investigation, Methodology, Supervision, Writing – review & editing M. R. Stavseth: Conceptualization, Formal analysis, Investigation, Methodology, Supervision, Writing – review & editing S. T. Bogstrand: Conceptualization, Methodology, Supervision, Writing – review & editing T. Tverborgvik: Conceptualization, Formal analysis, Methodology, Supervision, Writing – review & editing. All authors have approved the final article.

Patient consent for publication: not required.

Ethics approval

The study was approved by the Regional Committees for Medical and Health Research Ethics, Region South-East Norway, reference number 2012/140 and the Norwegian Directorate for Correctional Services. The study was approved exempt from informed consent. The linkage was performed by the Norwegian Institute of Public Health, who prepared the files for analysis.

Data availability: This population study was based on individual-level data from The Norwegian Prison Registry (held by the Directorate of Norwegian Correctional Service) and the Norwegian Cause of Death Register (held by the Norwegian Institute of Public Health). The ethical approval of this research project does not include permission to publicly share the raw data.

For peer review only - http://bmjopen.bmj.com/site/about/guidelines.xhtml

References

1. Valen A, Bogstrand ST, Vindenes V, *et al.* Driver-related risk factors of fatal road traffic crashes associated with alcohol or drug impairment. Accid Anal Prev. 2019;131:191-9. doi: 10.1016/j.aap.2019.06.014
2. Walsh JM, Flegel R, Atkins R, *et al*. Drug and alcohol use among drivers admitted to a Level-1 trauma center. Accid Anal Prev. 2005;37(5):894-901. doi: 10.1016/j.aap.2005.04.013
3. Bogstrand ST, Larsson M, Holtan A, *et al*. Associations between driving under the influence of alcohol or drugs, speeding and seatbelt use among fatally injured car drivers in Norway. Accid Anal Prev. 2015;78:14-9. doi: 10.1016/j.aap.2014.12.025
4. Bernhoft IM, Hels T, Lyckegaard A, *et al*. Prevalence and Risk of Injury in Europe by Driving with Alcohol, Illicit Drugs and Medicines. Procedia Soc Behav Sci. 2012;48:2907-16. doi: 10.1016/j.sbspro.2012.06.1259
5. Christophersen AS, Mørland J, Stewart K, *et al*. International trends in alcohol and drug use among vehicle drivers. Forensic science review. 2016;28(1):37-66. PMID: 26841722
6. Jamt REG, Gjerde H, Romeo G, *et al*. Association between alcohol and drug use and arrest for driving under the influence after crash involvement in a rural area of Norway: a case-control study. BMJ Open. 2019;9(1):e023563. doi: 10.1136/bmjopen-2018-023563
7. Oksanen A, Aaltonen M, Kivivuori J. Driving under the influence as a turning point? A register-based study on financial and social consequences among first-time male offenders. Addiction. 2015;110(3):471-8. doi: 10.1111/add.12777
8. Skurtveit S, Christophersen AS, Grung M, *et al*. Increased mortality among previously apprehended drunken and drugged drivers. Drug Alcohol Depend. 2002;68(2):143-50. doi: 10.1016/s0376-8716(02)00185-0
9. Skardhamar T, Skirbekk V. Relative Mortality among Criminals in Norway and the Relation to Drug and Alcohol Related Offenses. PloS One. 2013;8(11):e78893. doi: 10.1371/journal.pone.0078893
10. Impinen A, Mäkelä P, Karjalainen K, *et al*. High mortality among people suspected of drunk-driving. An 18-year register-based follow-up. Drug Alcohol Depend. 2010;110(1-2):80-4. doi: 10.1016/j.drugalcdep.2010.02.007
11. Gjerde H, Mørland J. Concentrations of carbohydrate-deficient transferrin in dialysed plasma from drunken drivers. Alcohol Alcohol. 1987;22(3):271-6. PMID: 2887174
12. Gjerde H, Sakshaug J, Mørland J. Heavy Drinking among Norwegian Male Drunken Drivers: A Study of γ-Glutamyltransferase. Alcohol Clin Exp Res. 1986;10(2):209-12. doi: 10.1111/j.1530-0277.1986.tb05073.x
13. Ruud J, Gjerde H, Mørland J. Alcohol consumption among convicted drunken drivers in Norway. Journal of traffic medicine. Tidsskr Nor Laegeforen.1993;21(3):121-5. PMID: 1462298
14. Brinkmann B, Beike J, Köhler H, *el al*. Incidence of alcohol dependence among drunken drivers. Drug Alcohol Depend. 2002;66(1):7-10. doi: 10.1016/s0376-8716(01)00177-6
15. Impinen A, Mäkelä P, Karjalainen K, *et al*. The Association between Social Determinants and Drunken Driving: A 15-Year Register-based Study of 81,125 Suspects. Alcohol Alcohol. 2011;46(6):721-8. doi: 10.1093/alcalc/agr075
16. Ministry of Transport. *Lov om vegtrafikk (LOV-1965-06-18-4) [Road traffic act].* Oslo, Norway: Ministry of Transport; 2012. Available from [Lov om vegtrafikk (vegtrafikkloven) - Lovdata] (accessed 12 January 2023)

For peer review only - http://bmjopen.bmj.com/site/about/guidelines.xhtml

17. Vindenes V, Jordbru D, Knapskog AB, *et al*. Impairment based legislative limits for driving under the influence of non-alcohol drugs in Norway. Forensic Sci Int. 2012;219(1-3):1-11. doi: 10.1016/j.forsciint.2011.11.004
18. Ministry of Transport. *Forskrift om påvirkning av andre rusmidler enn alkohol (FOR-2012-01-20-85) [Regulations on fixed limits for impairment by other intoxicating or anaesthetic/sedative substances than alcohol etc.]*. Oslo, Norway: Ministry of Transport; 2022. Available from Forskrift om faste grenser for påvirkning av andre berusende eller bedøvende middel enn alkohol m.m - Lovdata (accessed 12 January 2023)
19. Furuhaugen H, Jamt REG, Nilsson G, *et al*. Roadside survey of alcohol and drug use among Norwegian drivers in 2016-17: a follow up of the 2008-9 survey. Traffic Inj Prev. 2018:1-32. doi: 10.1080/15389588.2018.1478087
20. Gjerde H, Christophersen AS, Normann PT, *et al*. Norwegian roadside survey of alcohol and drug use by drivers (2008-2009). Traffic Inj Prev. 2013;14(5):443-52. doi: 10.1080/15389588.2012.728016
21. Gjerde H, Frost J. Prevalence of alcohol and drugs among drivers killed in road traffic crashes in Norway during 2011–2020. Traffic Inj Prev. 2023:1-6. doi: 10.1080/15389588.2023.2174801
22. Valen A, Bogstrand ST, Vindenes V, *et al*. Toxicological findings in suspected drug-impaired drivers in Norway — Trends during 1990–2015. Forensic Sci Int. 2017;280:15-24. doi: 10.1016/j.forsciint.2017.09.010
23. Gjerde H, Bogstrand ST, Lillsunde P. Commentary: Why Is the Odds Ratio for Involvement in Serious Road Traffic Accident Among Drunk Drivers in Norway and Finland Higher Than in Other Countries? Traffic Inj Prev. 2014;15(1):1-5. doi: 10.1080/15389588.2013.780651
24. Ulleberg P. Personality subtypes of young drivers. Relationship to risk-taking preferences, accident involvement, and response to a traffic safety campaign. Transp Res Part F Traffic Psychol Behav. 2001;4(4):279-97. https://doi.org/10.1016/S1369-8478(01)00029-8
25. Fair H, Walmsley R. World Prison Population List. Institute for Crime & Justice Policy Research; 2021. Available from: world_prison_population_list_13th_edition.pdf (prisonstudies.org) (accessed 11 May 2022)
26. Bukten A, Stavseth MR. Suicide in prison and after release: a 17-year national cohort study. Eur J Epidemiol. 2021;36(10):1075-83. doi: 10.1007/s10654-021-00782-0
27. The Norwegian Correctional Services. Annual Statistics 2020. Lillestrøm; 2021. Available from: Årstatistikk+2020+-+Endelig+KDI.pdf (accessed 6 April 2022)
28. The Norwegian Correctional Services. 2020 Annual Report. Lillestrøm; 2021. Available from: arsrapport_kdi_2020.pdf (regjeringen.no) (accessed 6 April 2022)
29. Bukten A, Lokdam NT, Clausen T, *et al*. Use and possession of illegal substances and driving while intoxicated among Norwegian prison inmates 2000-2019. Norwegian Centre for Addiction Research; 2021. Available at: seraf-rapport-3-2021-bruk-besittelse-og-ruspavirket-kjoring-.pdf (uio.no)
30. Binswanger IA, Stern MF, Deyo RA, *et al*. Release from prison--a high risk of death for former inmates. N Engl J Med. 2007;356(2):157-65. doi: 10.1056/NEJMsa064115
31. Graham L, Fischbacher CM, Stockton D, *et al*. Understanding extreme mortality among prisoners: a national cohort study in Scotland using data linkage. Eur J Public Health. 2015;25(5):879-85. doi: 10.1093/eurpub/cku252
32. Zlodre J, Fazel S. All-cause and external mortality in released prisoners: systematic review and meta-analysis. AmJ Public Health. 2012;102(12):e67-75. doi: 10.2105/AJPH.2012.300764
33. Kariminia A, Butler T, Corben S, *et al*. Extreme cause-specific mortality in a cohort of adult prisoners—1988 to 2002: a data-linkage study. Int Epidemiol. 2006;36(2):310-6. doi: 10.1093/ije/dyl225
34. Norwegian Centre for Addiction Research. The Norwegian Prison Release Study - nPRIS 2023. Available from: https://en.prisud.no/npris (accessed 12 January 2023)

For peer review only - http://bmjopen.bmj.com/site/about/guidelines.xhtml

35. Pedersen AG, Ellingsen CL. Data quality in the Causes of Death Registry. Tidsskr Nor Laegeforen. 2015;135(8):768-70. doi: 10.4045/tidsskr.14.1065
36. The Directorate of e-health. The ICD-10 (and ICD-11) coding system. Available from: https://www.ehelse.no/kodeverk-terminologi/kodeverket-icd-10-og-icd-11 (accessed 23 September 2022)
37. Statistics Norway. Offences and victims reported to the police 2022. Available from: https://www.ssb.no/en/sosiale-forhold-og-kriminalitet/kriminalitet-og-rettsvesen/statistikk/anmeldte-lovbrudd-og-ofre (accessed 11 January 2023)
38. Fazel S, Seewald K. Severe mental illness in 33,588 prisoners worldwide: systematic review and meta-regression analysis. Br J Psychiatry. 2012;200(5):364-73. doi: 10.1192/bjp.bp.111.096370
39. Fazel S, Yoon IA, Hayes AJ. Substance use disorders in prisoners: an updated systematic review and meta-regression analysis in recently incarcerated men and women. Addiction. 2017;112(10):1725-39. doi: 10.1111/add.13877
40. Karjalainen K, Lintonen T, Impinen A, *et al*. Mortality and causes of death among drugged drivers. J Epidemiol Community Health. 2010;64(6):506-12. doi: 10.1136/jech.2009.096990
41. Arries EJ, Maposa S. Cardiovascular Risk Factors Among Prisoners: An Integrative Review. J Forensic Nurs. 2013;9(1):52-64. doi: 10.1097/JFN.0b013e31827a59ef
42. Herbert K, Plugge E, Foster C, *et al*. Prevalence of risk factors for non-communicable diseases in prison populations worldwide: a systematic review. Lancet. 2012;379(9830):1975-82. doi: 10.1016/S0140-6736(12)60319-5
43. Skurtveit S, Christophersen AS, Beylich KM, *et al*. Study of rearrests for drunken driving in Norway. Forensic Sci Int. 1998;92(1):21-8. doi: 10.1016/s0379-0738(97)00199-0
44. Impinen A, Rahkonen O, Karjalainen K, *et al*. Substance use as a predictor of driving under the influence (DUI) rearrests. A 15-year retrospective study. Traffic Inj Prev. 2009;10(3):220-6. doi: 10.1080/15389580902822725
45. LaBrie RA, Kidman RC, Albanese M, *et al*. Criminality and continued DUI offense: criminal typologies and recidivism among repeat offenders. Behav Sci Law. 2007;25(4):603-14. doi: 10.1002/bsl.769
46. C'de Baca J, Miller WR, Lapham S. A multiple risk factor approach for predicting DWI recidivism. J Subst Abuse Treat. 2001;21(4):207-15. doi: 10.1016/s0740-5472(01)00204-5
47. Bishop NJ. Predicting rapid DUI recidivism using the Driver Risk Inventory on a state-wide sample of Floridian DUI offenders. Drug Alcohol Depend. 2011;118(2):423-9. doi: 10.1016/j.drugalcdep.2011.05.006
48. Holmgren A, Holmgren P, Kugelberg FC, *et al*. High re-arrest rates among drug-impaired drivers despite zero-tolerance legislation. Accid Anal Prev. 2008;40(2):534-40. doi: 10.1016/j.aap.2007.08.009
49. Zuckerman M. Sensation Seeking: Behavioral Expressions and Biosocial Bases. In: Wright JD, editor. International Encyclopedia of the Social & Behavioral Sciences (Second Edition). Oxford: Elsevier; 2015. p. 607-14.
50. Sarramon C, Verdoux H, Schmitt L, *et al*. Addiction and personality traits: sensation seeking, anhedonia, impulsivity]. Encephale. 1999;25(6):569-75. PMID: 10668599
51. Roth M, Schumacher J, Brähler E. Sensation seeking in the community: Sex, age and sociodemographic comparisons on a representative German population sample. Pers Individ Dif. 2005;39(7):1261-71. doi: 10.1016/j.paid.2005.05.003
52. Nordentoft M, Wahlbeck K, Hällgren J, *et al*. Excess mortality, causes of death and life expectancy in 270,770 patients with recent onset of mental disorders in Denmark, Finland and Sweden. PloS One. 2013;8(1):e55176. doi: 10.1371/journal.pone.0055176

For peer review only - http://bmjopen.bmj.com/site/about/guidelines.xhtml

53. Spittal MJ, Forsyth S, Borschmann R, *et al*. Modifiable risk factors for external cause mortality after release from prison: a nested case–control study. Epidemiol Psychiatri Sci. 2019;28(2):224-33. doi: 10.1017/S2045796017000506

54. Tverborgvik T, Stavseth MR, Bukten A. The association between drug use and mortality in a norwegian prison cohort: a prospective cohort study. Health Justice. 2023;11(1):22. doi: 10.1186/s40352-023-00223-y

55. Kariminia A, Law MG, Butler TG, *et al*. Factors associated with mortality in a cohort of Australian prisoners. Eur J Epidemiol. 2007;22(7):417-28. doi: 10.1007/s10654-007-9134-1

56. Bukten A, Herskedal A, Skurtveit S, *et al*. Driving under the influence (DUI) among patients in opioid maintenance treatment (OMT): a registry-based national cohort study. Addiction. 2013;108(11):1954-61. doi: 10.1111/add.12275

57. Barta WD, Fisher V, Hynes P. Decreased re-conviction rates of DUI offenders with intensive supervision and home confinement. Am J Drug Alcohol Abuse. 2017;43(6):742-6. doi: 10.1080/00952990.2016.1237519

58. Miller PG, Curtis A, Sønderlund A, *et al*. Effectiveness of interventions for convicted DUI offenders in reducing recidivism: a systematic review of the peer-reviewed scientific literature. Am J Drug Alcohol Abuse. 2015;41(1):16-29. doi: 10.3109/00952990.2014.966199

59. World Health Organization. Status report on prison health in the WHO European Region. Copenhagen: WHO Regional Office for Europe; 2019.

For peer review only

For peer review only - http://bmjopen.bmj.com/site/about/guidelines.xhtml

Supplementary tables

Supplementary table 1. Mean age at death (with standard deviation) for all causes of death

Cause of death	No DUI	DUI		
		DUI only	DUI drug	DUI other
Cancer	58.87 (10.93)	62.97 (9.29)	58.90 (10.64)	61.87 (9.23)
Circulatory	56.70 (12.55)	60.80 (10.77)	57.02 (12.24)	59.18 (10.63)
Respiratory	58.99 (11.33)	63.55 (10.43)	60.67 (13.55)	63.50 (12.72)
Digestive	57.08 (10.82)	56.82 (7.59)	54.00 (11.90)	54.00 (8.41)
Alcoholic liver disease	52.12 (7.91)	56.53 (7.24)	54.46(7.92)	52.31 (5.49)
Other internal	51.47 (14.18)	60.25 (11.53)	51.76 (13.74)	60.90 (15.27)
**Total internal causes**	**56.57 (12.23)**	**61.12 (10.20)**	**56.95 (12.13)**	**59.57 (10.93)**
Transport-related	36.62 (11.40)	49.54 (7.98)	38.33 (12.09)	38.47 (12.38)
Intoxication[1]	38.45 (11.38)	48.49 (13.23)	40.27 (12.02)	46.21 (13.23)
Alcohol-related	54.08 (9.53)	57.89 (9.39)	54.96 (11.81)	54.61 (10.25)
Drug-related	36.59 (10.08)	40.56 (10.54)	37.44 (9.80)	39.54 (11.48)
Suicide	37.51 (11.30)	42.78 (14.65)	37.37 (9.75)	41.32 (12.89)
Homicide	36.19 (11.06)	-	41.00 (9.68)	-
Other accident-related	45.14 (14.84)	50.62 (11.91)	47.08 (14.88)	49.97 (15.60)
**Total external causes**	**38.69 (11.93)**	**47.75 (13.32)**	**40.38 (12.22)**	**45.11 (13.88)**
Unexplained	50.94 (13.25)	58.34 (11.99)	53.90 (12.81)	53.00 (12.02)
Unknown cause	47.21 (13.32)	55.72 (12.99)	48.52 (13.55)	54.73 (10.81)
**Total deceased**	**46.42 (14.86)**	**56.39 (13.03)**	**47.62 (14.63)**	**52.77 (14.12)**

[1] Alcohol- and drug-related combined

For peer review only

Table 4. Logistic regression for all-causes, natural causes and unnatural causes of death. Estimates given as crude (cOR) and adjusted odds ratios (aOR) with corresponding 95% confidence intervals. The level of statistical significans was $p \leq 0.05$

	**All cause of death, n=8053**				**Natural causes of death, n=3379**				**Unatural causes of death, n=4139**			
	cOR		aOR[1]		cOR		aOR[1]		cOR		aOR[1]	
Sex (female ref.)	1.21 (1.12-1.31)	**<0.001**	1.22 (1.12-1.32)	**<0.001**	1.20 (1.07-1.36)	**0.003**	1.18 (1.04-1.33)	**0.011**	1.16 (1.04-1.29)	**0.009**	1.11 (1.00-1.24)	**0.063**
Age[2]	1.06 (1.05-1.06)	**<0.001**	1.06 (1.05-1.06)	**<0.001**	1.11 (1.10-1.11)	**<.001**	1.11 (1.10-1.11)	**<.001**	1.01 (1.01-1.02)	**<.001**	1.01 (1.01-1.02)	**<.001**
No. of convictions	1.03 (1.01-1.04)	**<0.001**	0.99 (0.97-1.00)	**0.092**	0.88 (0.86-0.91)	**<0.001**	0.85 (0.83-0.88)	**<0.001**	1.09 (1.08-1.11)	**<0.001**	1.06 (1.04-1.08)	**<0.001**
Conviction (no dui ref.):	-		-		-		-		-		-	
DUI-only	1.28 (1.18-1.38	**<.001**	0.99 (0.91-1.07)	**0.916**	1.91 (1.72-2.11)	**<.001**	1.11 (0.99-1.24)	**0.066**	0.78 (0.69-0.89)	**<.001**	0.78 (0.69-0.89)	**<.001**
DUI-drug	1.59 (1.51-1.69)	**<.001**	1.51 (1.44-1.63)	**<.001**	1.61 (1.47-1.75)	**<.001**	1.80 (1.64-1.98)	**<.001**	1.60 (1.49-1.73)	**<.001**	1.45 (1.34-1.57)	**<.001**
DUI-other	1.42 (1.26-1.59)	**<.001**	1.23 (1.09-1.38)	**<.001**	1.72 (1.46-2.03)	**<.001**	1.34 (1.12-1.60)	**0.001**	1.16 (0.98-1.37)	**0.095**	1.13 (0.95-1.34)	**0.164**

[1]Adjusted for covariates significant in crude analyses
[2]Age at last incarceratinon

For peer review only

For peer review only

STROBE Statement—Checklist of items that should be included in reports of ***cohort studies***

	Item No	Recommendation	Page No
**Title and abstract**	1	(*a*) Indicate the study's design with a commonly used term in the title or the abstract<br>(*b*) Provide in the abstract an informative and balanced summary of what was done and what was found	1-3
**Introduction**			
Background/rationale	2	Explain the scientific background and rationale for the investigation being reported	4-5
Objectives	3	State specific objectives, including any prespecified hypotheses	5
**Methods**			
Study design	4	Present key elements of study design early in the paper	6
Setting	5	Describe the setting, locations, and relevant dates, including periods of recruitment, exposure, follow-up, and data collection	6
Participants	6	(*a*) Give the eligibility criteria, and the sources and methods of selection of participants. Describe methods of follow-up<br>(*b*) For matched studies, give matching criteria and number of exposed and unexposed	6-7
Variables	7	Clearly define all outcomes, exposures, predictors, potential confounders, and effect modifiers. Give diagnostic criteria, if applicable	7-8
Data sources/ measurement	8*	For each variable of interest, give sources of data and details of methods of assessment (measurement). Describe comparability of assessment methods if there is more than one group	6-7
Bias	9	Describe any efforts to address potential sources of bias	8
Study size	10	Explain how the study size was arrived at	6
Quantitative variables	11	Explain how quantitative variables were handled in the analyses. If applicable, describe which groupings were chosen and why	8-9
Statistical methods	12	(*a*) Describe all statistical methods, including those used to control for confounding<br>(*b*) Describe any methods used to examine subgroups and interactions<br>(*c*) Explain how missing data were addressed<br>(*d*) If applicable, explain how loss to follow-up was addressed<br>(*e*) Describe any sensitivity analyses	8-9
**Results**			
Participants	13*	(a) Report numbers of individuals at each stage of study—eg numbers potentially eligible, examined for eligibility, confirmed eligible, included in the study, completing follow-up, and analysed<br>(b) Give reasons for non-participation at each stage<br>(c) Consider use of a flow diagram	6,9
Descriptive data	14*	(a) Give characteristics of study participants (eg demographic, clinical, social) and information on exposures and potential confounders<br>(b) Indicate number of participants with missing data for each variable of interest<br>(c) Summarise follow-up time (eg, average and total amount)	10
Outcome data	15*	Report numbers of outcome events or summary measures over time	10-11

For peer review only - http://bmjopen.bmj.com/site/about/guidelines.xhtml

Main results	16	(*a*) Give unadjusted estimates and, if applicable, confounder-adjusted estimates and their precision (eg, 95% confidence interval). Make clear which confounders were adjusted for and why they were included<br>(*b*) Report category boundaries when continuous variables were categorized<br>(*c*) If relevant, consider translating estimates of relative risk into absolute risk for a meaningful time period	12-13
Other analyses	17	Report other analyses done—eg analyses of subgroups and interactions, and sensitivity analyses	-
**Discussion**			
Key results	18	Summarise key results with reference to study objectives	14
Limitations	19	Discuss limitations of the study, taking into account sources of potential bias or imprecision. Discuss both direction and magnitude of any potential bias	16
Interpretation	20	Give a cautious overall interpretation of results considering objectives, limitations, multiplicity of analyses, results from similar studies, and other relevant evidence	14-16
Generalisability	21	Discuss the generalisability (external validity) of the study results	16
**Other information**			
Funding	22	Give the source of funding and the role of the funders for the present study and, if applicable, for the original study on which the present article is based	18

*Give information separately for exposed and unexposed groups.

Note: An Explanation and Elaboration article discusses each checklist item and gives methodological background and published examples of transparent reporting. The STROBE checklist is best used in conjunction with this article (freely available on the Web sites of PLoS Medicine at http://www.plosmedicine.org/, Annals of Internal Medicine at http://www.annals.org/, and Epidemiology at http://www.epidem.com/). Information on the STROBE Initiative is available at http://www.strobe-statement.org.

For peer review only
